# Development of Resistance to Clarithromycin and Amoxicillin-Clavulanic Acid in *Lactiplantibacillus plantarum In Vitro* Is Followed by Genomic Rearrangements and Evolution of Virulence

**DOI:** 10.1128/spectrum.02360-21

**Published:** 2022-05-17

**Authors:** V. V. Kostenko, A. A. Mouzykantov, N. B. Baranova, E. A. Boulygina, M. I. Markelova, D. R. Khusnutdinova, M. V. Trushin, O. A. Chernova, V. M. Chernov

**Affiliations:** a Laboratory of Molecular Bases of Pathogenesis, Kazan Institute of Biochemistry and Biophysics, Russian Academy of Sciences, Kazan, Russia; b Institute of Fundamental Medicine and Biology, Kazan Federal University, Kazan, Russia; Regional Centre for Biotechnology

**Keywords:** probiotics, *Lactiplantibacillus plantarum*, antibiotic resistance, genomic rearrangements, mobilome, resistome, virulome, phenotypic resistance, virulence, *Drosophila melanogaster*

## Abstract

Ensuring the safety of the use of probiotics is a top priority. Obviously, in addition to studying the beneficial properties of lactic acid bacteria, considerable attention should be directed to assessing the virulence of microorganisms as well as investigating the possibility of its evolution under conditions of selective pressure. To assess the virulence of probiotics, it is now recommended to analyze the genomes of bacteria in relation to the profiles of the virulome, resistome, and mobilome as well as the analysis of phenotypic resistance and virulence *in vitro*. However, the corresponding procedure has not yet been standardized, and virulence analysis of strains *in vivo* using model organisms has not been performed. Our study is devoted to testing the assumption that the development of antibiotic resistance in probiotic bacteria under conditions of selective pressure of antimicrobial drugs may be accompanied by the evolution of virulence. In this regard, special attention is required for the widespread in nature commensals and probiotic bacteria actively used in pharmacology and the food industry. As a result of step-by-step selection from the Lactiplantibacillus plantarum 8p-a3 strain isolated from the “Lactobacterin” probiotic (Biomed, Russia), the *L. plantarum* 8p-a3-Clr-Amx strain was obtained, showing increased resistance simultaneously to amoxicillin-clavulanic acid and clarithromycin (antibiotics, the combined use of which is widely used for Helicobacter pylori eradication) compared to the parent strain (MIC_8p-a3-Clr-Amx_ of 20 μg/mL and 10 μg/mL, and MIC_8p-a3_ of 0.5 μg/mL and 0.05 μg/mL, respectively). The results of a comparative analysis of antibiotic-resistant and parental strains indicate that the development of resistance to the corresponding antimicrobial drugs in *L. plantarum in vitro* is accompanied by the following: (i) significant changes in the genomic profile (point mutations as well as deletions, insertions, duplications, and displacement of DNA sequences) associated in part with the resistome and mobilome; (ii) changes in phenotypic sensitivity to a number of antimicrobial drugs; and (iii) an increase in the level of virulence against Drosophila melanogaster, a model organism for which *L. plantarum* is considered to be a symbiont. The data obtained by us indicate that the mechanisms of adaptation to antimicrobial drugs in *L. plantarum* are not limited to those described earlier and determine the need for comprehensive studies of antibiotic resistance scenarios as well as the trajectories of virulence evolution in probiotic bacteria *in vivo* and *in vitro* to develop a standardized system for detecting virulent strains of the corresponding microorganisms.

**IMPORTANCE** Ensuring the safety of the use of probiotics is a top priority. We found that increased resistance to popular antimicrobial drugs in Lactiplantibacillus plantarum is accompanied by significant changes in the genomic profile and phenotypic sensitivity to a number of antimicrobial drugs as well as in the level of virulence of this bacterium against *Drosophila*. The data obtained in our work indicate that the mechanisms of antibiotic resistance in this bacterium are not limited to those described earlier and determine the need for comprehensive studies of the potential for the evolution of virulence in lactic acid bacteria *in vivo* and *in vitro* and to develop a reliable control system to detect virulent strains among probiotics.

## INTRODUCTION

Ecology and evolution of virulence of organisms are closely interrelated. Any changes in the environment (related to climate, host population density, restriction of food resources, selective pressure of antimicrobials, etc.) can affect the adaptability of bacteria and the evolution (development) of bacterial virulence (the appearance of virulence in harmless environmental microbes and tritagonists [commensals and symbionts]) or a change in the degree of virulence in pathogenic microorganisms (its increase [progression], weakening [regression], or disappearance) (1, 2).

The data obtained in recent years testifying to a variety of sophisticated ways of bacterial survival under conditions of selective antibiotic pressure associated with multiple, including large-scale, changes in the genomic profile by noncanonical mechanisms and unpredictable trajectories of virulence evolution determine the need to revise our ideas about the possibilities of adaptation of microbes to stressors and conduct detailed studies of antibiotic resistance scenarios in pathogenic and nonpathogenic bacteria under different environmental conditions *in vivo* and *in vitro* to develop a global control system for the emergence and spread of new types of pathogens. The number of reports that detail the development of antibiotic resistance under conditions of selective pressure in commensals and are accompanied not only by point mutations of target proteins but also by large-scale genomic rearrangements associated with the resistome and mobilome as well as the evolution of virulence is growing (3–8). In this regard, special attention is required for the widespread in nature commensals and probiotic bacteria actively used in pharmacology and the food industry (9). Ensuring the safety of the use of probiotics is a top priority task (10, 11). There is no doubt that in addition to studying the beneficial properties of lactic acid bacteria, considerable attention should be directed to assessing the virulence of microorganisms as well as investigating the possibility of its evolution under conditions of selective pressure of antimicrobials (12). To assess the virulence of probiotics, it is now recommended to analyze the genomes of bacteria in relation to the profiles of the virulome, resistome, and mobilome as well as the analysis of phenotypic resistance and virulence *in vitro* (3, 13, 14). However, the corresponding procedure has not yet been standardized, and the virulence analysis of strains *in vivo* using model organisms has not been performed.

Earlier (15), in a model of the ubiquitous commensal bacterium Acholeplasma laidlawii, which is a representative of a taxon (class *Mollicutes*) phylogenetically close to lactobacilli, we showed that even bacteria associated with the smallest prokaryotes capable of independent reproduction, like classical bacteria (16, 17), may use more than one adaptation scenario to one antimicrobial drug, and the development of antibiotic resistance is accompanied by changes in their genomic profile and virulence *in vitro* (8) and *in vivo* (15). The virulence analysis of *A. laidlawii in vivo* was performed in Drosophila melanogaster, an organism used for a wide range of model studies, in relation to which representatives of the *Mollicutes* class can be commensals and/or pathogens (18). Conducting similar studies with respect to probiotic bacteria, which are the basic residents of the intestinal microbiota of higher organisms, including D. melanogaster, seems very relevant. However, there are no such works yet.

The present work is devoted to verifying the assumption that the development of antibiotic resistance in probiotic bacteria under conditions of selective pressure may be accompanied by the evolution of virulence. The study was conducted on a model of *Lactiplantibacillus plantarum*, one of the most studied species widely used in the food industry as a probiotic microorganism and/or microbial starter culture. As a result of step-by-step selection from the *L. plantarum* 8p-a3 strain isolated from the Lactobacterin probiotic, the *L. plantarum* 8p-a3-Clr-Amx strain was obtained and showed increased resistance compared with the parent strain to amoxicillin-clavulanic acid (MIC of 20 μg/mL) and clarithromycin (MIC of 10 μg/mL), antibiotics widely used for Helicobacter pylori eradication (19, 20), and a comparative analysis of the genomic profile (including virulome, mobilome, and resistome), phenotypic resistance to antibiotics of different groups, and virulence of the corresponding strains against D. melanogaster, a model organism for which *L. plantarum* is considered to be a symbiont, was performed.

## RESULTS

### Main characteristics of *L. plantarum* strains with differential sensitivity to antibiotics.

**FIG 1:**
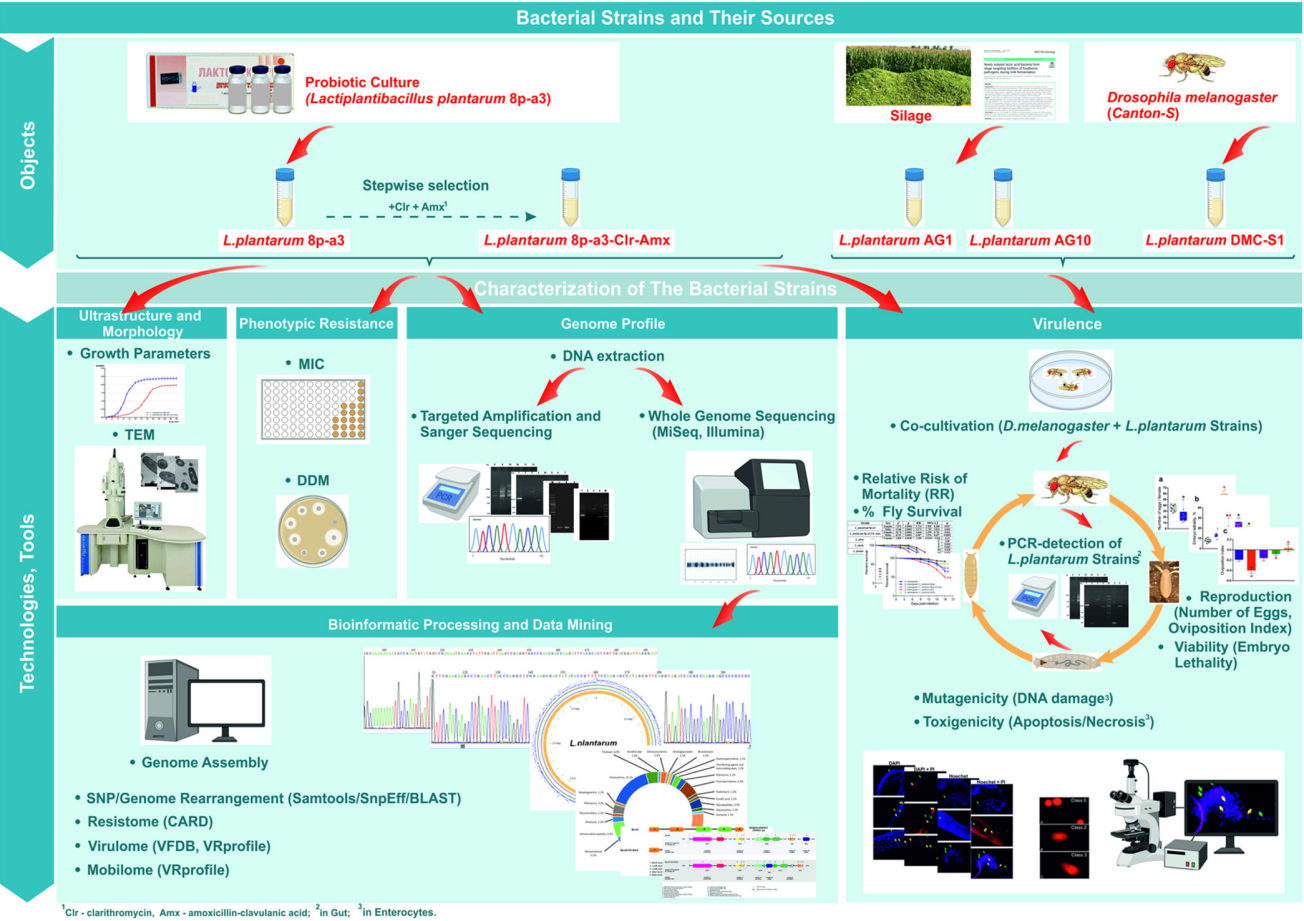
Graphical representation of the workflow; TEM, transmission electron microscopy; DDM, disk diffusion method.

As a result of step-by-step selection from the strain *L. plantarum* 8p-a3 isolated from the probiotic “Lactobacterin,” we obtained the strain *L. plantarum* 8p-a3-Clr-Amx, which showed increased resistance to amoxicillin-clavulanic acid and clarithromycin simultaneously (MIC values of 20 μg/mL and 10 μg/mL, respectively; Fig. 1). The physiological and morphological characteristics of the cultures of the corresponding strains are presented in Fig. S1, S2, and S9 and Table S1 in the supplemental material. The cells of the original and antimicrobial-resistant strains of *L. plantarum* do not significantly differ in size (*P* > 0.05; lengths of 1,415 ± 299 nm and 1,225 ± 215 nm and widths of 580 ± 48 nm and 533 ± 71 nm in 8p-a3 and 8p-a3-Clr-Amx, respectively; Fig. S9). Meanwhile, we found that the adaptation of *L. plantarum* 8p-a3 to antibacterial drugs is accompanied by a change in the morphology of their colonies. Cells of the parent strain 8p-a3 form smooth, rounded, or slightly elongated colonies on MRS medium, whereas cells of the resistant strain 8p-a3-Clr-Amx form smaller, rough colonies (Fig. S1). The appearance of rough colonies in bacteria is associated with excessive synthesis of extracellular matrix components, which additionally protects cells from the action of antibacterial drugs (21). The formation of small colonies has been described in *L. plantarum* WCFS1 during adaptation to stressful conditions (22).

Unlike the parent strain, *L. plantarum* 8p-a3-Clr-Amx is characterized by a longer lag phase, and the stationary phase has a lower value in terms of optical density. The specific growth rate of the antibiotic-resistant strain is lower (0.387 ± 0.01 h^−1^) than that of the parent strain (0.745 ± 0.035 h^−1^), and the generation time is longer (1.792 ± 0.046 versus 0.932 ± 0.044 h; Fig. S2 and Table S1). According to the reported data, antibiotic-resistant bacteria, compared with wild-type bacteria, may have both a slower and an increased specific growth rate (23, 24). This phenomenon may be associated with the fitness cost of adaptation, and most often the adaptation of bacteria to antibiotics is accompanied by a significant decrease in the growth rate of the culture (25). Morphological differences of colonies as well as the difference in growth parameters in *L. plantarum* 8p-a3 and *L. plantarum* 8p-a3-Clr-Amx indicate differences in strains of biochemical processes associated with, inter alia, replication and cell division, which may be mediated by the differential primary structure of some genes and/or their expression.

### Genomic profiles of *L. plantarum* 8p-a3 and *L. plantarum* 8p-a3-Clr-Amx strains.

The whole-genome sequences of *L. plantarum* 8p-a3 and *L. plantarum* 8p-a3-Clr-Amx were determined by us using the MiSeq platform (Illumina, USA) and submitted to the GenBank database (https://www.ncbi.nlm.nih.gov/bioproject/, accession number PRJNA528387). Using the software tools SAMtools, SnpEff, and BLAST, we conducted a comparative analysis of the genomes of these strains (as well as *L. plantarum* strains presented in the Virulence Factor Database (VFDB), Comprehensive Antibiotic Resistance Database (CARD), and VRprofile databases) and inventoried their resistomes (Table S2), mobilomes (Tables S3 and S4), and virulomes (Tables S5 and S6).

As a result of a comparative analysis of the genomic profiles of the strains, we found changes in the sequences of 18 open reading frames (ORFs) as well as one intergenic region of *L. plantarum* 8p-a3-Clr-Amx (Table 1). Changes in the genome of *L. plantarum* 8p-a3-Clr-Amx were associated with both point mutations and larger-scale genomic rearrangements. Single substitutions were detected in a number of genes (*E3U93_01740* G212A, *E3U93_00235* 1183delT, *E3U93_02280* C117A, *E3U93_06520* C668A, *E3U93_07190* T1275G, *E3U93_08315* T224G, *E3U93_08755* G902A, *E3U93_08955* C667T, *E3U93_14460* A1039G, and an intergenic region in the contig SOQA01000025.1 A13292C); however, in some, multiple changes (*E3U93_10425* G766A, A799C, A800C, C801A, A802T, G816C, C819G, G820A, C843T, G847A, G853A, A874G, and C886T; *E3U93_12775* A177G, C321T, T324C, A328G, and C358T; *E3U93_01760* 277 to 282del; *E3U93_RS08405* 291 to 362del; and *E3U93_10790* 97 to 135del) and/or major rearrangements (deletion of 1,456 nucleotides [positions 128709 to 130164] from the contig SOQA01000003.1, deletion of 102 nucleotides [positions 1 to 102] from the contig SOQA01000016.1; *E3U93_04390* transposase insertion into the gene) were detected (Fig. S3 and S4). At the same time, mutations associated with the virulome, resistome, and mobilome turned out to be single (1, 1, and 2, respectively). It was revealed that the development of resistance in *L. plantarum* is accompanied by the insertion of the transposase gene (ISLpL3 family transposase, E3U93_16150 gene locus) into the esterase gene (locus E3U93_04390). The large-scale genomic rearrangement has highlighted the possibility of creating a probe for differential detection of *L. plantarum* 8p-a3 and *L. plantarum* 8p-a3-Clr-Amx, which was designed and used in our work to control D. melanogaster infection with the corresponding strains in a comparative analysis of their virulence (Fig. S5).

**TABLE 1:** Changes in the primary structure of the *L. plantarum* 8p-a3-Clr-Amx genome relative to *L. plantarum* 8p-a3

No.	Gene/protein ID^*a*^	Protein^*b*^^,^^*c*^	EggNog^*d*^	Protein function	Changes in genome^*e*^
1	E3U93_01740/TFE52705.1	50S ribosomal protein L4 **<R>**	J	Participates in the assembly of the 50S ribosome subunit. It stimulates the binding of ribosomal protein L22 with 23S rRNA.	G212A
2	E3U93_01760/TFE52709.1	50S ribosomal protein L22 **<R>**	J	Participates in the assembly of the 50S ribosome subunit. Specifically binds to 23S rRNA.	Deletion of 6 nucleotides 277–282
3	E3U93_00235/TFE52434.1	Bifunctional lysylphosphatidylglycerol flippase/synthetase MprF **{R}<R><V>**	M	Catalyzes the modification of phosphatidylglycerin/cardiolipin (negatively charged membrane lipids) with l-lysine as well as the translocation of aminoacylphosphatidylglycerin from the inner surface of the membrane to the outer. Such modification is important in the development of resistance to cationic antimicrobial peptides.	Deletion of T at position 1183
4	E3U93_02280/TFE51920.1	Transcription termination factor Rho **<R><V>**	K	Rho factor involved in transcription termination.	C117A
5	E3U93_04390/TFE51650.1	Esterase	I	Catalyzes the hydrolysis of esters into alcohols and acids. Substrate specificity has not been determined. It is assumed to participate in lipid metabolism.	Embedding a transposase inside a gene
6	E3U93_06520/TFE51083.1	PhoH family protein **{M}<R>**	T	ATPase. It is induced by phosphorus starvation. Presumably involved in signal transduction.	C668A
7	E3U93_07190/TFE50703.1	Penicillin-binding protein 2 **<R><V>**	M	The protein contains the FtsI domain. FtsI is an essential cell division protein that synthesizes peptidoglycan of the cell wall in the septa region. FtsI has transglycosylase and transpeptidase activities. It has penicillin-binding properties.	T1275G
8	E3U93_08315/TFE50913.1	CDP-glycerol-polyglycerophosphate glycerophosphotransferase	M	Participates in the synthesis of poly-glycerophosphate of teichoic acids of the bacterial cell wall.	T224G
9	E3U93_RS08405/WP_166783984.1	Bacterial Ig-like domain-containing protein **<V>**	M	The function is not defined. Bacterial proteins containing an Ig-like domain are involved in conjugation, adhesion, biofilm formation, folding, and secretion.	Deletion of 72 nucleotides 291–362
10	E3U93_08755/TFE50541.1	Phosphodiesterase of the DHF family **<R><V>**	T	Hydrolyzes cyclic di-AMP, which regulates various cellular pathways involved in stress response, biofilm formation, cell wall homeostasis, antibiotic resistance, and expression of bacterial virulence factors.	G902A
11	E3U93_08955/TFE50579.1	Response regulator YycF **<R><V>**	T	Regulation of gene expression.	C667T
12	E3U93_10425/TFE50187.1	Peptidoglycan endopeptidase	M	Hydrolyzes the peptidoglycan of the bacterial cell wall during growth and division.	G766A, A799C, A800C, C801A, A802T, G816C, C819G, G820A, C843T, G847A, G853A, A874G, and C886T
13	E3U93_10790/TFE49954.1	50S ribosomal protein L32	J	Structural protein of the 50S ribosome subunit. The function is not clear.	Deletion of 39 nucleotides 97–135
14	E3U93_12775/TFE49354.1	DUF4428 domain-containing protein	S	Function is not clear.	A177G (A1293G)^*f*^, C321T (C1437T), T324C (T1440C), A328G (A1444G), and C358T (C1474T)
15	E3U93_14460/TFE48645.1	PASTA domain-containing protein **<R><V>**	M	The protein contains two separate domains: FtsI and PASTA. FtsI is an essential cell division protein that synthesizes peptidoglycan of the cell wall in the septa region. FtsI has transglycosylase and transpeptidase activities. It has penicillin-binding properties. PASTA domain interacts with an unrelated peptidoglycan and spatially brings it closer to the peptidoglycan biosynthesis complex; also has penicillin-binding properties.	A1039G
16	Contig SOQA01000025.1	Intergenic region			A13292C
17	E3U93_16165/TFE47830.1	NlpC/P60 family protein **<R><V>**	M	The family of proteins containing the NlpC/P60 domain includes acyltransferases, amylases, and endopeptidases. Endopeptidases hydrolyze peptide bonds in the cell wall. Proteins are involved in cell division, maintaining the integrity of the cell wall. Some proteins of the family are immunogenic and are necessary for the realization of virulence.	A lot of changes
18	E3U93_04615/TFE51695.1 and E3U93_04620/TFE51696.1	LysM peptidoglycan-binding domain-containing protein and SdpI family protein {M}**<R><V>**	ST	The function of a protein with a LysM domain is not clear. Specifically interacts with sugars of the bacterial cell wall. Two more domains were found in the protein, presumably involved in cell wall degradation and invasion. The protein of the SdpI family is a membrane protein and participates in signal transduction. It protects the bacterial cell from toxins produced by it.	Deletion of 1,456 nucleotides (position on contig 128709–130164)
19	E3U93_13185/pseudo^*g*^	MucBP domain-containing protein **<V>**	M	Participates in adhesion, and binds specifically to mucin.	Deletion of 102 nucleotides (position on contig 1–102)

a Gene locus and protein ID by annotation of *L. plantarum* strain 8p-a3.

b {R}, {M}, the corresponding proteins belong to the resistome and mobilome, respectively (according to CARD and VRprofile).

c **<R>**, **<V>**, homologous genes in other bacteria are associated with the development of antibiotic resistance and the realization of virulence, respectively.

d Functional categories are specified according to EggNog; I, lipid transport and metabolism; J, translation, ribosome structure, and biogenesis; K, transcription; M, cell wall/membrane/shell biogenesis; S, function unknown; T, mechanisms of signal transduction.

e Change in the genome of *L. plantarum* strain 8p-a3-Clr-Amx.

f The gene has not been fully sequenced in strains 8p-a3 and 8p-a3-Clr-Amx. The numbering of the positions of nucleotide substitutions is indicated by strain 8p-a3 and in parentheses by strain 8P-A3 (NZ_CP046726), the genome of which is fully (complete) sequenced.

g The gene is not fully sequenced, and, therefore, it is annotated as a pseudogene. Two fragments of the gene are localized in different contigs, SOQA01000016.1 and SOQA01000031.1.

Nucleotide sequences corresponding to the 18 mutant ORFs encode proteins of different functional classes (Table 1), of which only one (bifunctional lysylphosphatidylglycerol flippase/MprF synthetase), according to the CARD database (Table S3 and Fig. S6), is associated with *Lactobacillus* resistance, but the contribution of the corresponding gene and the product encoded by it to resistance to clarithromycin and amoxicillin-clavulanic acid is not known. Meanwhile, changes in some genes mutated in *L. plantarum* 8p-a3-Clr-Amx are associated (according to the reported data) in different bacterial species with changes in sensitivity to antimicrobial drugs of different groups and the level of virulence of microorganisms (Table 1). To find out whether the corresponding changes occur in the case of *L. plantarum*, we conducted a comparative analysis of phenotypic resistance and virulence of strains 8p-a3 and 8p-a3-Clr-Amx.

### Phenotypic antibiotic resistance profiles in *L. plantarum* 8p-a3 and *L. plantarum* 8p-a3-Clr-Amx strains.

To determine the phenotypic resistance of *L. plantarum* 8p-a3 and *L. plantarum* 8p-a3-Clr-Amx to antimicrobial drugs of different groups, we applied an approach based on the assessment of MIC by microdilution (26) and also used the disk diffusion method (27). According to our comparative analysis of the profiles of phenotypic antibiotic resistance of *L. plantarum* strains (Table 2), the development of resistance to clarithromycin and amoxicillin-clavulanic acid in the lactic acid bacterium may be accompanied by a change in sensitivity to antimicrobial drugs of different groups, the development of resistance to fluoroquinolones (ofloxacin) and cephalosporins (cefazolin, ceftazidime), but a decrease in resistance to aminoglycosides (gentamicin), tetracyclines (tetracycline), and rifampicin.

**TABLE 2:** Profiles of phenotypic antibiotic resistance in *L. plantarum* 8p-a3 and *L. plantarum* 8p-a3-Clr-Amx strains

Antibiotic	Strain^*a*^							
	*L. plantarum* 8p-a3			*L. plantarum* 8p-a3-Clr-Amx				
	DDM^*b*^	Reaction to the antibiotic^*c*^	MIC (mcg/mL)	DDM^*b*^	Reaction to the antibiotic^*c*^	MIC (mcg/mL)	Cut-off value (mg/L)^*d*^	AR^*e*^
Amikacin	8.6 ± 0.5/30	R	256 ± 0.0	14.5 ± 0.5/30	R	32 ± 0.0	ND	+
**Amoxicillin**	**20.3 ± 1.6/20**	**S**	**0.05 ± 0.0**	**12 ± 1.7/20**	**R**	**20 ± 0.0**	ND	**+**
Ampicillin	20.5 ± 1.3/10	S	2 ± 0.0	5/10	R	32 ± 0.0	2.0	+
Vancomycin	9.1 ± 0.8/30	R	>256	6.5 ± 0.1/30	R	>256	NR	+
Gentamicin	8.4 ± 0.6/10	R	NT	15 ± 0.2/10	S	NT	16.0	+
Imipenem	25.5 ± 1.3/10	S	NT	20.5 ± 0.9/10	S	NT	ND	+
Kanamycin	5.2 ± 0.6/30	R	>256	10 ± 0.6/30	R	125	64.0	+
**Clarithromycin**	**22 ± 1.6/15**	**S**	**0.5 ± 0.0**	**11.8 ± 0.5/15**	**R**	**10 ± 0.0**	ND	**+**
Clindamycin	22.5 ± 1.2/2	S	NT	31.5 ± 1.1/2	S	NT	4.0	+
Linezolid	20.5 ± 0.8/30	S	NT	25.5 ± 0.8/30	S	NT	ND	−
Meropenem	18.6 ± 0.8/10	S	NT	26.5 ± 0.4/10	S	NT	ND	+
Norfloxacin	5.1 ± 0.2/10	R	NT	5.5 ± 0.2/10	R	NT	ND	+
Ofloxacin	12.5 ± 1.6/5	I	NT	10.5 ± 1.2/5	R	NT	ND	+
Penicillin	NT	NT	>256	NT	NT	>256	ND	+
Rifampicin	14.5 ± 1.1/5	R	NT	21.5 ± 1.6/5	S	NT	ND	+
Streptomycin	5.3 ± 0.3/30	R	32 ± 0.0	12.5 ± 0.4/30	I	32 ± 0.0	NR	+
Tetracycline	14.5 ± 0.9/30	R	125 ± 0.0	25 ± 1.1/30	S	32 ± 0.0	32.0	+
Trimethoprim/Sulfamethoxazole	22.4 ± 0.4/1.25/23.75	S	NT	24 ± 2/1.25/23.75	S	NT	ND	−
Cefazolin	16.6 ± 1.1/30	I	NT	12.5 ± 1.1/30	R	NT	ND	+
Cefepime	12.5 ± 0.6/30	R	NT	9 ± 0.2/30	R	NT	ND	+
Cefoperazone	13 ± 1.1/75	R	NT	13.5 ± 0.3/75	R	NT	ND	+
Cefotaxime	10.6 ± 0.4/30	R	>256	10 ± 0.9/30	R	125 ± 0.0	ND	+
Ceftazidime	20 ± 1.2/30	S	NT	9 ± 0.2/30	R	NT	ND	+
Ceftriaxone	11.5 ± 0.7/30	R	125 ± 0.0	5 ± 0.1/30	R	>256	ND	+
Ciprofloxacin	5.2 ± 0.2/5	R	64 ± 0.0	11 ± 0.5/5	R	64 ± 0.0	NR	+
Erythromycin	18.5 ± 1.1/15	S	32 ± 0.0	10.5 ± 0.5/15	R	>256	1.0	+
Ertapenem	17.5 ± 0.6/10	I	NT	17 ± 0.7/10	I	NT	ND	+

a -, the strain *L. plantarum* 8p-a3 was isolated from the “Lactobacterin” probiotic (Biomed, Russia), the strain *L. plantarum* 8p-a3-Clr-Amx showing increased resistance simultaneously to amoxicillin-clavulanic acid and clarithromycin was obtained as a result of step-by-step selection from *L. plantarum* 8p-a3; bold formatting indicates sensitivity of the strains to amoxicillin and clarithromycin; NT, not tested.

b DDM, disk diffusion method (growth retardation zone, mm/concentration, mcg/disk).

c The reaction to the antibiotic determined by the disk diffusion method; S, sensitive; I, intermediate; R, resistant.

d Microbiological cutoff values for antibiotics for *L. plantarum*, as provided by the European Food Safety Authority (EFSA) 2012 guideline.

e AR, proteins associated with antibiotic resistance (based on CARD; see Table S2 in the supplemental material); ND, no data; NR, not required. Because the mutant strain has a delayed growth phases (Fig. S2), MIC has been evaluated at different time points according to the growth phases of the strains.

In some cases, the data obtained by us on the phenotypic resistance of *L. plantarum* strains differ significantly from the data of the *in silico* analysis. For example, according to phenotypic resistance data, strain 8p-a3 is resistant to amikacin, vancomycin, gentamicin, kanamycin, norfloxacin, rifampicin, streptomycin, tetracycline, cefepime, cefoperazone, cefotaxime, ceftriaxone, and ciprofloxacin, and strain 8p-a3-Clr-Amx is resistant to amikacin, amoxicillin, ampicillin, vancomycin, kanamycin, clarithromycin, norfloxacin, ofloxacin, cefazolin, cefepime, cefoperazone, cefotaxime, ceftazidime, ceftriaxone, ciprofloxacin, and erythromycin (Table 2); however, according to genomic data, this is not the case (corresponding specific mutations or genes are missing, or appropriate nucleotide sequences have a low percentage of similarity; Table S2).

### Virulence of *L. plantarum* strains.

**FIG 2:**
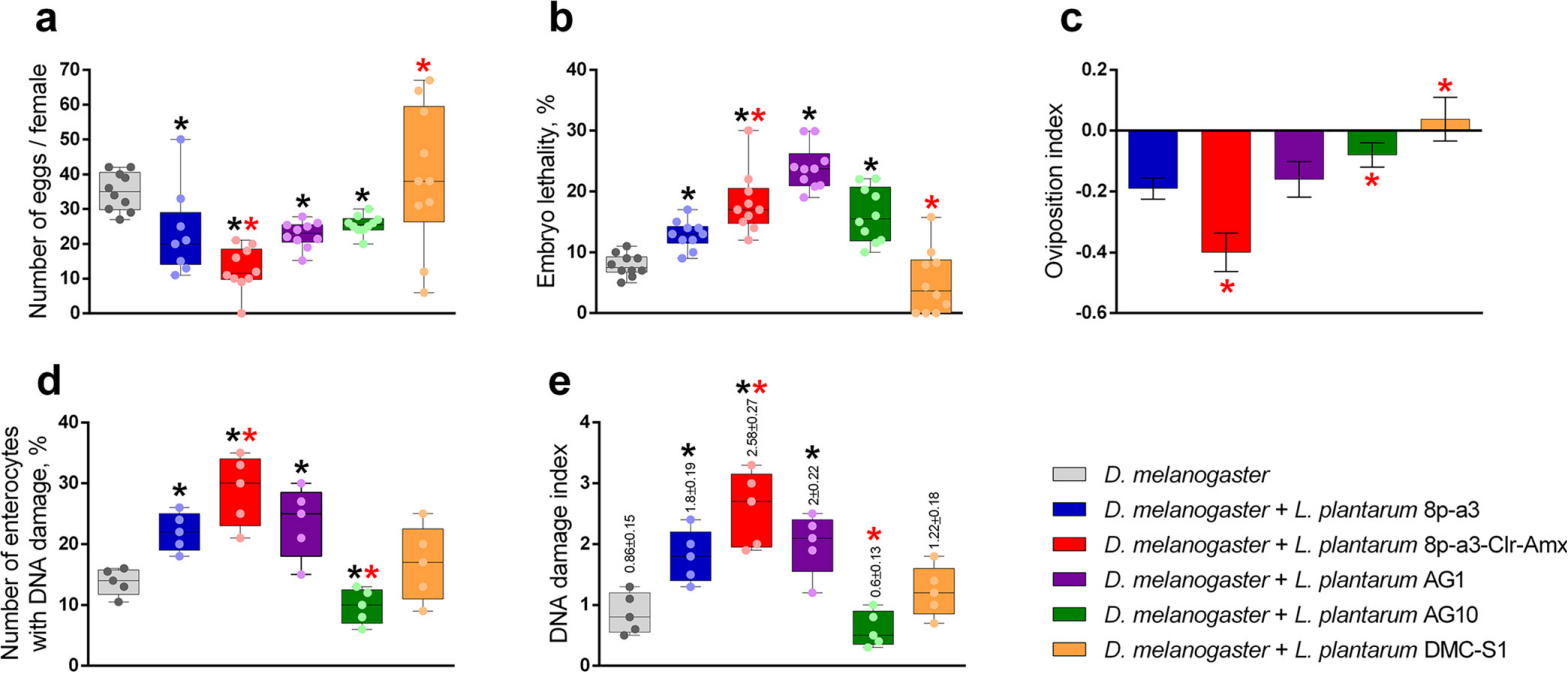
Influence of *L. plantarum* on reproduction parameters, viability, and enterocytes of D. melanogaster. (a to c) Indicators characterizing the reproductive potential of *Drosophila*. (d and e) Indicators that allow assessment of the virulence of *L. plantarum* with respect to the genome integrity of *Drosophila* enterocytes. Each spot shows an independent sample. The box and whisker plots show the average, 75% quartiles, and extremes values. The DNA damage index (e) was calculated using the formula (0 × *n*_0_ + 1 × *n*_1_ + 2 × *n*_2_ + 3 × *n*_3_ + 4 × *n*_4_)/*Σ*, where *n*_0_ to *n*_4_ are the numbers of DNA comets of each type, and *Σ* is the sum of the analyzed DNA comets. The values were compared with each other using one-factor analysis of variance (one-way ANOVA) using a Bonferroni *post hoc* test; *, *P* < 0.05 compared to the group of uninfected flies; red *, *P* < 0.05 compared to the group of flies infected with the strain *L. plantarum* 8p-a3.

**FIG 3:**
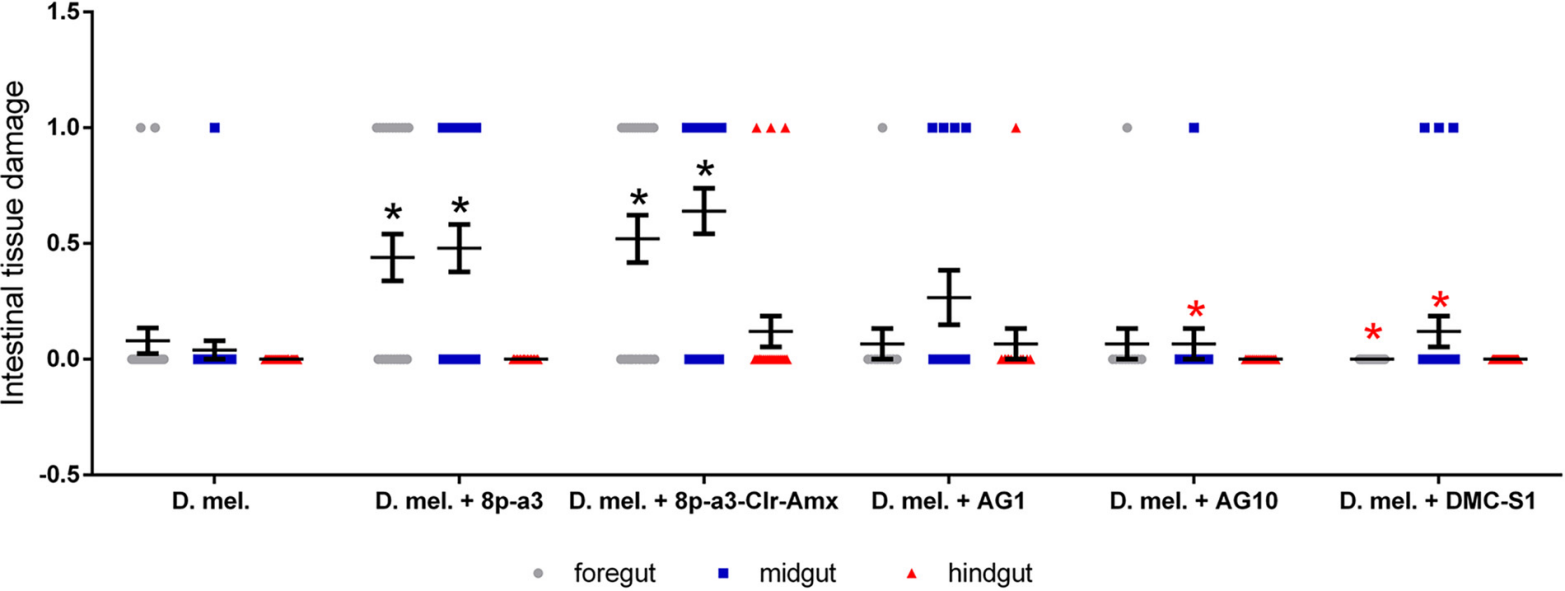
Changes in the intestinal tissues of D. melanogaster associated with infection with *L. plantarum* strains. Intestinal lesions of larvae were detected in the anterior, middle, and posterior sections by staining with trypan blue (*n* = 25 for each group). The values were compared with each other using one-factor analysis of variance (one-way ANOVA) using a Bonferroni *post hoc* test; *, *P* < 0.05 compared to the group of uninfected flies; red *, *P* < 0.05 compared to the group of flies infected with the strain *L. plantarum* 8p-a3.

Virulence analysis of *L. plantarum* strains was performed on D. melanogaster, an organism in relation to which *L. plantarum* is considered to be a symbiont. Control of flies for infection with the studied strains was carried out using specific primers, providing differential detection of *L. plantarum* 8p-a3 and *L. plantarum* 8p-a3-Clr-Amx (Fig. S5) due to different patterns of PCR amplicons of the nucleotide sequences of the gene encoding esterase (locus E3U93_04390) in the strains. In flies, the standard indicators used to assess virulence (viability and reproduction) were determined. To do this, we analyzed the number of eggs laid, embryonic death, and egg laying index. In addition, taking into account the data of Fast et al. (28), we also evaluated the toxigenicity and genotoxicity of the strains against the intestinal tissue of flies. With this aim, we analyzed the number of enterocytes of flies with DNA damage and the index of DNA comets (IDC). The results of the studies are presented in Fig. 2 and 3 and in Fig. S7 and S8.

According to the data obtained, both strains adversely affect the studied parameters of D. melanogaster, but *L. plantarum* 8p-a3-Clr-Amx shows a more pronounced negative effect than the original (parent) strain. Thus, infection of D. melanogaster with *L. plantarum* 8p-a3 and *L. plantarum* 8p-a3-Clr-Amx strains leads to a decrease in the number of eggs laid (Fig. 2). Significant differences were found both in comparison with the uninfected group (by 33% [*P* < 0.005] and 62% [*P* < 0.0001], respectively) and between strains (the resistant strain has a more pronounced virulence than the original strain [*P* < 0.007]). Infection of D. melanogaster with *L. plantarum* strains is associated with an increase in the number of dead individuals at the embryonic stage of development (Fig. 2). Significant differences were found both in comparison with the uninfected group (infection with *L. plantarum* 8p-a3 increases the embryonic death of flies by 30% [*P* < 0.004] and *L. plantarum* 8p-a3-Clr-Amx by 51% [*P* < 0.0013]) and between strains (infection of *Drosophila* with a resistant strain in comparison with the parent strain increases the embryonic mortality of flies by 29.5% [*P* < 0.017]).

Infection of D. melanogaster larvae with *L. plantarum* 8p-a3 and *L. plantarum* 8p-a3-Clr-Amx strains is associated with a significant increase in intestinal tissue damage compared to the uninfected group (Fig. 3 and Fig. S7). The *L. plantarum* strain 8p-a3 causes an increase in the number of lesions by 36% (*P* < 0.05) in the anterior intestine and by 44% (*P* < 0.05) in the middle intestine compared to the intact group. The strain resistant to clarithromycin and amoxicillin-clavulanic acid had a more pronounced negative effect; in flies infected with this strain, compared with the control group, the number of lesions in the anterior part increased by 44% (*P* < 0.05) and in the middle part of the intestine by 56% (*P* < 0.05). In addition, in flies infected with *L. plantarum* 8p-a3-Clr-Amx, in contrast to flies infected with strain 8p-a3, lesions were also found in the posterior intestine in 8% of cases (*P* < 0.05; Fig. 3).

*Drosophila* infection with the *L. plantarum* strains was associated with an increase in single-strand DNA breaks in enterocytes (Fig. 2 and Fig. S8). Significant differences were found compared with the control group (*L. plantarum* 8p-a3 increases the number of enterocytes with DNA damage by 1.6-fold [*P* < 0.02; IDC = 1.8 ± 0.19] and *L. plantarum* 8p-a3-Clr-Amx by 2.1-fold [*P* < 0.007; IDC = 2.58 ± 0.27]), and between strains, the antibiotic-resistant strain turned out to be more aggressive in this case (*P* < 0.04).

Infection with *L. plantarum* 8p-a3 and *L. plantarum* 8p-a3-Clr-Amx strains affects the survival and relative mortality risk of *Drosophila* flies (Fig. S10 and Table S8), and the antibiotic-resistant strain compared to the original strain of *L. plantarum* is more aggressive (*χ*^2^ of 42.01 and *P* < 0.0001 and *χ*^2^ of 13.66 and *P* < 0.0002 for males; *χ*^2^ of 16.70 and *P* < 0.0001 and *χ*^2^ of 7.675 and *P* < 0.0056 for females, respectively; relative mortality risk [RR] of 5.6667 and 95% confidence interval [95% CI] of 2.9518 to 10.8784 and RR of 3.8889 and 95% CI of 1.9740 to 7.6614 for males; RR of 3.8333 and 95% CI of 2.4726 to 5.9430 and RR of 2.9444 and 95% CI of 1.8639 to 4.6514 for females, respectively). An RR value above 1.0 indicates a higher risk of death under the influence of these infections.

Thus, according to our results, the development of resistance in *L. plantarum* to two antibiotics at once is accompanied by an increase in the virulence level of the lactic acid bacterium against D. melanogaster. Because the original strain 8p-a3 showed virulence against *Drosophila*, we also conducted appropriate studies for additional strains of *L. plantarum*, including AG1 and AG10 (isolated from silage and characterized in terms of probiotic potential [29]) and the strain of *L. plantarum* DMC-S1 (isolated by us from the resident gut microbiota of D. melanogaster *Canton-S*).

It has been found that infection of D. melanogaster with *L. plantarum* AG1 and *L. plantarum* AG10 strains leads to a decrease in the number of eggs laid (Fig. 2). Significant differences were found in comparison with the uninfected group (by 48% [*P* < 0.005] and 28% [*P* < 0.0001], respectively). Infection of D. melanogaster with *L. plantarum* DMC-S1 has no significant effect on egg production (*P* > 0.05).

Infection of D. melanogaster with strains of *L. plantarum* AG1, AG10, or DMC-S1 did not lead to significant (*P* > 0.05) damage to the intestines of flies (Fig. 3 and Fig. S7). At the same time, infection of *Drosophila* with *L. plantarum* AG1, but not with *L. plantarum* AG10, is associated with an increase in single-stranded DNA breaks in enterocytes (Fig. 2 and Fig. S8). Significant differences were found compared with the control group (*L. plantarum* AG1 increases the number of enterocytes with DNA damage by 1.7-fold [*P* < 0.05; IDC = 2 ± 0.22], whereas *L. plantarum* AG10 reduces the number of enterocytes with DNA damage by 1.4-fold [*P* < 0.05; IDC = 0.6 ± 0.13]). Infection of D. melanogaster with *L. plantarum* DMC-S1 does not lead to significant changes in single-stranded DNA breaks in enterocytes (*P* > 0.05).

Infection with *L. plantarum* AG1 and *L. plantarum* AG10 strains affects the survival and relative mortality risk of *Drosophila* (*χ*^2^ of 7.837 and *P* = 0.0051 and *χ*^2^ of 4.436 and *P* = 0.0352 for males; *χ*^2^ of 2.805 and *P* = 0.0940 and *χ*^2^ of 4.728 and *P* = 0.0297 for females, respectively; RR of 3.0000 and 95% CI of 1.4873 to 6.0512 and RR of 1.6667 and 95% CI of 0.7651 to 3.6305 for males; RR of 2.3333 and 95% CI of 1.4474 to 3.7616 and RR of 2.6667 and 95% CI of 1.6743 to 4.2472 for females, respectively; Fig. S10 and Table S8). An RR value above 1.0 indicates a higher risk of death under the influence of these infections. However, in the case of the strain DMC-S1 (*χ^2^* of 5.825 and *P* = 0.0158 and *χ^2^* of 3.973 and *P* = 0.0462; RR of 0.5556 and 95% CI of 0.1930 to 1.5996 and RR of 0.3889 and 95% CI of 0.1699 to 0.8900 for males and females, respectively), this turned out not to be the case. An RR value below 1.0 indicates a lower risk of death of flies under the influence of this infection (Fig. S10 and Table S8).

To find out whether the effects of the strains studied on the physical parameters of *Drosophila* are independent/dependent on the growth advantage accumulated by these strains, a quantitative estimation of bacterial growth in terms of CFU was done (Fig. S11 and Table S9).

It was found that the growth parameters significantly differ among the strains studied. The highest specific growth rate was in 8p-a3 (0.745 ± 0.035 h^−1^), and the lowest was in 8p-a3-Clr-Amx (0.387 ± 0.01 h^−1^). The longest generation time was in 8p-a3-Clr-Amx (1.792 ± 0.046 h), and the shortest was in 8p-a3 (0.932 ± 0.044 h). The difference in the specific growth rate and generation time in AG1 and DMC-S1 (virulent and avirulent strains, respectively) did not reach reliability (*P* > 0.05). At the same time, there were no significant differences between *L. plantarum* strains in CFU at different stages of bacterial growth (Fig. S11; *P* > 0.05).

The maximum specific growth rate (*μ*_max_) and lag time (LT) are considered to be the two most important parameters of microbial dynamics, which can reflect the growth advantages of bacterial strains, fitting, and virulence (30). Short LT and/or high *μ*_max_ generally present a positive correlation with virulence of bacterial strains; however, in our work, we have found no such trend (Fig. S11B and Table S9).

As shown in Table S9, the significant differences in maximum growth rates and lag time between strains were observed. The highest value of LT was in 8p-a3-Clr-Amx (10 ± 0.4 h), and the lowest was in 8p-a3 (3.6 ± 0.2). The highest values of *μ*_max_ were in the avirulent strain DMC-S1 (1.029 ± 0.034 h^−1^) that did not show virulence against D. melanogaster and in the control strain AG10 (0.958 ± 0.06 h^−1^) that showed negative effects on *Drosophila*. The lowest value of *μ*_max_ was in 8p-a3-Clr-Amx (0.581 ± 0.009 h^−1^), the strain that showed the most pronounced virulence against fruit flies. Among the control strains that showed negative effects in *Drosophila* (AG1, AG10, and 8p-a3), significant differences in *μ* and *λ* were also observed. At that, the lowest *μ*_max_ and the highest lag time were found in the AG1 strain (0.791 ± 0.053 and 5.2 ± 0.3 versus 0.958 ± 0.061 and 4.8 ± 0.2 in AG10, 0.949 ± 0.027 and 3.6 ± 0.2 in 8p-a3, respectively, *P* = 0.0001), whose negative effects on D. melanogaster were close to AG10 and 8p-a3 strains (Table S8).

## DISCUSSION

Our study is devoted to testing the assumption that the development of antibiotic resistance in probiotic bacteria under conditions of selective pressure of antimicrobial drugs may be accompanied by the evolution of virulence. The analysis was performed by us on a model of *Lactiplantibacillus plantarum*, one of the most studied species widely used in the food industry as a probiotic microorganism and/or microbial starter culture. As a result of step-by-step selection from the *L. plantarum* 8p-a3 strain isolated from the “Lactobacterin” probiotic (Biomed, Russia), the *L. plantarum* 8p-a3-Clr-Amx strain was obtained, showing increased resistance simultaneously to amoxicillin-clavulanic acid and clarithromycin (antibiotics, the combined use of which is widely used for H. pylori eradication) compared to the parent strain (MIC_8p-a3-Clr-Amx_ of 20 μg/mL and 10 μg/mL and MIC_8p-a3_ of 0.5 μg/mL and 0.05 μg/mL, respectively). The results of a comparative analysis of antibiotic-resistant and parental strains indicate that the development of resistance to the corresponding antimicrobial drugs in *L. plantarum in vitro* is associated with multiple changes in the genomic profile of the bacterium.

However, none of the mutations identified by us in the genome of *L. plantarum* 8p-a3-Clr-Amx were previously described as the root cause, that is, obligately determining the occurrence of resistance to appropriate antibiotics in lactobacilli. In principle, the results of the active application of genomic profiling to determine the molecular scenarios of antibiotic resistance in different bacteria *in vitro* and *in vivo* in the last decade have made it possible to verify that the genetic signatures of antibiotic resistance are not always valid; phenotypic resistance in bacteria is not always accompanied by mutations in the genes of antimicrobial targets (8, 31, 32). Our results indicate that *L. plantarum* can complement the list of similar cases. The data obtained in our work indicate that the mechanisms of antibiotic resistance in this bacterium are not limited to those described earlier, and current ideas about the possibilities of adaptation of *L. plantarum* to antimicrobial drugs need revision.

Phenotypic antibiotic resistance profiles in *L. plantarum* 8p-a3 and *L. plantarum* 8p-a3-Clr-Amx revealed in our study demonstrate that the development of resistance to clarithromycin and amoxicillin-clavulanic acid in the lactic acid bacterium may be accompanied by a change in sensitivity to antimicrobial drugs of different groups. The effects may reflect the fitness cost and may partly be mediated by changes in the *L. plantarum* genome, affecting, among other things, enzyme genes and structural target proteins for antimicrobials. This assumption may be supported by data obtained with respect to some other bacteria. For example, substitution (G70D) in ribosomal protein L4 in Neisseria gonorrhoeae is associated with the development of resistance to clarithromycin and erythromycin (33), and one of the substitutions (T345I, L776S, A475P, L459_H466 del, L826F, L826F, L826F, T345A, L291I, L291I, W424R, L341S, and S337L) in the MprF protein in Staphylococcus aureus isolates is associated with the development of resistance to vancomycin (34); deletion of the *rho* gene in Escherichia coli causes an increase in cell sensitivity to rifampicin and gentamicin (35), and substitutions (A311V, I312M, V316T, V316P, T483S, F504L, N512Y, and G545S) in penicillin-binding protein 2 were found in ceftriaxone-resistant strains of Neisseria gonorrhoeae (36).

The exact contribution of mutations in the corresponding *L. plantarum* genes to the change in the sensitivity of the lactic acid bacterium to antibiotics of different groups has yet to be determined. Meanwhile, the presence of mutations in genes encoding proteins of various functional classes, including those involved in fundamental cellular processes, suggests the possibility of changing the metabolic capabilities of the bacterium, which determine, among other things, the status of virulence. This assumption is supported by literature data; changes in the primary structure of a number of genes that turned out to be mutant in *L. plantarum* 8p-a3-Clr-Amx led to changes in virulence in some bacteria. Thus, deletion of the gene encoding a protein with an immunoglobulin-like domain in Lactobacillus acidophilus led to a decrease in the virulent properties of the bacterium (37), deletion of the *rho* gene in Staphylococcus aureus led to an increase in the expression of virulence factors and an increase in the virulent properties of the bacterium against mice (38), and deletion of the *pbp2* gene in Erwinia amylovora led to the loss of virulence against plants (39). In this regard, to clarify the possibility of changing the virulence status in *L. plantarum* with the development of antibiotic resistance to amoxicillin-clavulanic acid and clarithromycin, we performed a comparative analysis of the virulence of strains 8p-a3 and 8p-a3-Clr-Amx.

According to the data obtained, both strains adversely affect the studied parameters (viability and reproduction of D. melanogaster) and show toxigenicity and genotoxicity against the intestinal tissue of flies, but *L. plantarum* 8p-a3-Clr-Amx shows a more pronounced negative effect than the original (parent) strain. Considering these results, that is, the presence of virulence in the original strain of *L. plantarum* and an increase in the degree of virulence in the antibiotic-resistant strain of *L. plantarum*, it can be concluded that the development of resistance to two antibiotics in this bacterium is accompanied by a progression of virulence. This phenomenon may be partly due to the genomic rearrangements we have identified in the bacterium. The exact molecular mechanisms are yet to be determined. Meanwhile, the virulence of the original probiotic strain against *Drosophila* came as a surprise. Moreover, virulence was also shown by other *L. plantarum* strains isolated from silage (AG1 and AG10) but not the strain isolated from the resident microbiota of the *Drosophila* gut. And although the strains differed in the degree of negative impact and were significantly inferior to the resistant strain 8p-a3-Clr-Amx, the nature of their virulence requires explanation.

Since the critical elements of genomes from the point of view of assessing the safety of probiotic bacteria are the resistome, mobilome, and virulome, we paid special attention to the analysis of the corresponding modules in the studied strains of *L. plantarum* (antibiotic-resistant and original [parent] strains). According to *in silico* data, the genome of the parent strain of *L. plantarum* contains genes that determine the resistance of different bacteria to antimicrobial drugs of different classes (that is, genes that determine the resistome). But the level of similarity of gene sequences in the vast majority of cases does not exceed 50%. Only for 4 genes (encoding DNA-directed RNA polymerase subunit beta [TFE52697.1], ABC transporter ATP-binding protein [TFE51715.1], response regulator transcription factor [TFE48498.1], and ATP-binding cassette domain-containing protein [TFE51142.1]) the similarity of the sequences is 61.54, 56.18, 50.44, and 50.32%, respectively. However, these indicators are not significant, that is, allowing us to conclude that the probiotic strain may show resistance to antimicrobial drugs of the corresponding classes (rifamycin, lincosamides, glycopeptides, and tetracyclines). In this regard, for a correct conclusion about the sensitivity of probiotic strains to antimicrobial drugs, an analysis of phenotypic resistance is necessary. According to our analysis, in some cases, the data on the phenotypic resistance of *L. plantarum* strains differ significantly from the data of the *in silico* analysis (Table 2 and Table S2 in the supplemental material). This underlines the importance to supplement the genomic profiling data with appropriate phenotypic testing for the correct conclusion about the sensitivity of probiotic bacteria to antimicrobial drugs.

The presence of mobile elements (mobilome) in bacterial genomes determines the risk of lateral transfer of individual genes and/or large-scale rearrangements of the genome, which can cause significant changes in the properties of the bacterium, including the status of antibiotic resistance and virulence (40). In this regard, the determination of the safety status of probiotic bacteria includes an assessment of the risk of the development of relevant events *in silico* based on the analysis of mobile genetic elements in the genomic profile of bacteria. In the genome of the probiotic strain, we found prophage sequences and insertion sequence (IS) elements of different families as well as genes for integrases (phage integrase SAM-like domain-containing protein [Prophage_134287379], integrase/tyrosine-type recombinase/integrase [Prophage_157325322], integrase/tyrosine-type recombinase/integrase [Prophage_31415840], site-specific integrase [Prophage_157325260], site-specific integrase [Prophage_155042957], site-specific integrase [Prophage_28876262], site-specific integrase [Prophage_13095806], site-specific integrase [Prophage_22296542], site-specific integrase [Prophage_41179288], site-specific integrase [Prophage_48697280], and site-specific integrase [Prophage_13095681]) critical for lateral transfer and large-scale genomic rearrangements. These data indicate the existence of a risk of the development of relevant events in the probiotic strain of *L. plantarum*, especially under stressful conditions. The realization of a large-scale rearrangement associated with the insertion of the transposase gene (ISLpL3 family transposase, E3U93_16150 gene locus) into the *Lactobacillus* esterase gene was just recorded by us in an antibiotic-resistant strain. The results obtained by us indicate that under conditions of selective pressure of antimicrobial drugs, the features of the mobilome of the probiotic strain studied can determine large-scale genomic rearrangements. This fact compromises the safety status of the probiotic bacterium. Such mobile elements and related events can make a significant contribution to bacterial virulence and lead to an unpredictable chain of events in high-density microbial communities, for example, in the gut microbiome.

To date, a pool of critical genes that determine the virulence of *L. plantarum* strains has been determined; it includes 41 genes (41). These are *gelE* (gelatinase), *hyl* (hyaluronidase), *asa1* (aggregation substance), *esp* (enterococcal surface protein), *cylA* (cytolysin), *efaA* (endocarditis antigen), *ace* (adhesion of collagen), *vanA*, *vanB*, *vanC1*, *vanC2*, *vanC2/C3* (related to vancomycin resistance), *ermA*, *ermB*, *ermC* (related to erythromycin resistance), *tetK*, *tetL*, *tetM*, *tetO*, *tetS* (related to tetracycline resistance), *aac(6′)-Ie-aph(2”)-Ia* (related to gentamicin resistance), *aph(3′)-IIIa*, *ant(4′)-Ia*, *aph(2”)-Id*, *aph(2”)-Ic*, *aph(2”)-Ib*, *ant(6)-Ia* (related to aminoglycosides resistance), *catA* (chloramphenicol resistance), *bcrB*, *bcrD*, *bcrR* (related to bacitracin resistance), *ccf*, *cob*, *cpd* (related to sex pheromones), *sprE* (serine protease), *int*, *intTn* (transposon related), *hdc1*, *hdc2* (related to histidine decarboxylase), *tdc* (tyrosine decarboxylase), and *odc* (ornithine decarboxylase). In this regard, the analysis of the virulome module in the genome of a probiotic strain involves the detection of the corresponding genes. From this pool of genes in the genomes of the original and resistant strains of *L. plantarum*, we found only *int* genes encoding integrases. However, this fact alone, along with the case of genomic rearrangements recorded by us in a resistant strain, does not allow us to consider the original strain of *L. plantarum* absolutely safe. In addition, according to *in silico* data, the genome of the parent strain contains genes associated with virulence in a number of other bacteria. The similarity of the sequences of the corresponding genes ranges from 50% to 80%. At the same time, as part of the *L. plantarum* virulome (8p-a3 and 8p-a3-Clr-Amx), we discovered the *ndk* gene (TFE52116.1), the product of which (nucleoside diphosphate kinase [Ndk]) has recently become the object of close attention due to its pleiotropy in prokaryotes and eukaryotes, involvement in the interaction of micro- and macroorganisms, regulation of bacterial virulence, and the ability of this bacterial protein to induce single-stranded breaks in DNA in host cells (42). However, according to the results of a search in the GenBank database using the BLAST algorithm, this gene is also present in the genomes of most other *Lactobacillus* strains, and the results of our targeted testing indicate that it is also present in all the strains we studied (Fig. S5). It is possible that the strains differ in the level of its expression. At the same time, it is obvious that the difference in virulence of these strains is hardly limited to the differential expression of the only gene. In our studies, *L. plantarum* strains were found to be heterogeneous with respect to a number of growth parameters, but not CFU at control points. We focused on variation in lag times and max specific growth rates. Studies have shown that the length of the lag time can reflect the strain’s ability to respond to the new environment (43). High maximum growth rates generally present a positive correlation with virulence factors and pathogenicity (31). However, in the case of the strains studied by us, such a pattern was not traced. In relation to these indicators, the strains (including control ones that showed virulence against *Drosophila*) demonstrated heterogeneity. Moreover, the highest maximum growth rate was found in the avirulent strain, and the lowest maximum growth rate was found in the most aggressive antibiotic-resistant strain. These results and the data obtained by us regarding the differential sensitivity of D. melanogaster to *L. plantarum* strains isolated from different sources indicate that in the case of *L. plantarum*, the evaluation of strains by growth characteristics for the prediction of bacterial virulence may be ineffective. It is obvious that additional criteria and the search for molecular markers are required to assess the virulence potential of probiotic bacteria. The identification of a molecular signature that determines the virulence or avirulence of *L. plantarum* strains in relation to a specific host is a major challenge.

Bacterial virulence (“the relative capacity of a microorganism to cause damage in a host”) is a highly dynamic and context-dependent process (44, 45). The virulence of an infectious agent (the ability to damage the host during microbial infection [the acquisition of a microorganism by a host]) is the result of a complex network interaction of the signaling systems of a particular microorganism and its host in which the host microbiota is also involved (46). By themselves, host responses to a microorganism are known to have a damaging effect on host cells and tissues, and in some cases, they are the main cause of the severity of infection (45, 47). In this regard, it does not seem to be correct to look for the cause of virulence only in the features of the genomic profile and the pool of virulence factors in the *Lactobacillus* strain *in silico* and/or *in vitro*. It is obvious that in order to understand the molecular machinery of the nature of virulence *L. plantarum* will require comprehensive studies of the molecular mechanisms of interaction of different strains of *Lactobacillus* with different hosts, including different *Drosophila* lines, under different environmental conditions. Elucidation of these aspects is vital today for both fundamental studies of the effect of *L. plantarum* on the (neuro)physiology of the host and applied developments aimed at the use of these bacteria as probiotics.

The interactions of *L. plantarum* and D. melanogaster are currently in the zone of active attention both from the point of view of the fundamental foundations of the host-symbiont interaction and applied aspects related to the emerging possibilities of modulation of neurophysiology and reproduction of the host organism through probiotics (48–50). To date, as a result of studies of the relationship between D. melanogaster and its symbiont *L. plantarum*, various facets of micro- and macroorganism interaction have been discovered. It has been established that the lactic acid bacterium promotes larval growth (51, 52) and protein production (53, 54), regulates the host’s eating behavior (55–57), and also induces the generation of reactive oxygen species (ROS) by NADP oxidase (58) and protects fruit fly tissue cells from damaging agents (59). Sensational data were presented in the work of Rudman and coauthors (60), which demonstrated that the addition of *L. plantarum* to the nutrient medium of D. melanogaster induces a shift in the structure of the intestinal microbiota of *Drosophila* and rapid evolution in the fly population; significant changes in their genomic profile were detected in five generations. Another unexpected aspect of the interaction of the lactic acid bacterium with *Drosophila* was revealed in Fast et al. (28). In adult D. melanogaster individuals, monoassociation with *L. plantarum* (induced by the use of a cocktail of antibiotics to produce axenics followed by the use of ampicillin, metronidazole, vancomycin, and neomycin to maintain monoassociation and prevent infection with other bacteria) destroys intestinal homeostasis. The available facts about the relationship between *L. plantarum* and D. melanogaster indicate that the interactions of the bacterium and the host are complex and ambiguous. Our knowledge of these processes is still insufficient, and the lactic acid bacterium will surprise us more than once with the arsenal of self-defense tools and the spheres of its influence in relation to eukaryotic organisms.

The molecular mechanisms and conditions for the development of pathogenicity in lactic acid bacteria are of considerable interest both for fundamental studies of the trajectories of the evolution of virulence in commensals under different environmental conditions and practical developments related to the safety of the use of probiotic bacteria in the food and pharmaceutical industry as well as the reliability of the results obtained when using *Drosophila* in model scientific experiments. When studying D. melanogaster, the authors usually do not provide data on the genome profiles and phenotypic resistance of the *L. plantarum* strains used. It is obvious that these characteristics (along with some others, including the features of the microbiota structure of the used fly line) must be taken into account to minimize the mismatch of research results, which becomes a serious problem (61–63). Moreover, data on the transient versus resident bacterium strain may be quite different (as it was found in our study). This circumstance will also need to be taken into account in relevant studies.

### Conclusion.

The number of reports showing that the development of antibiotic resistance under conditions of selective pressure in commensals can be accompanied not only by point mutations of target proteins but also by large-scale genomic rearrangements associated with the resistome and mobilome as well as the evolution of virulence is growing (3–8). In this regard, the analysis of the genomes of probiotic bacteria with respect to the resistome, mobilome, and virulome is the focus of attention today (10, 13, 14, 64, 65), but systematic studies aimed at verifying the assumption regarding the evolution of virulence in these bacteria under conditions of selective antibiotic pressure are not yet available. We found that increased resistance to popular antimicrobial drugs in *L. plantarum* is accompanied by significant changes in the genomic profile and phenotypic sensitivity to a number of antimicrobial drugs as well as in the level of virulence of this bacterium against *Drosophila*. Recently, it has become clear that the arsenal of self-defense tools in bacteria can be inexhaustible (66), and there can be many adaptation scenarios even to one antimicrobial drug *in vitro* and *in vivo* (67, 68). To what extent this is true for *L. plantarum* remains to be seen. The data obtained in our work indicate gaps in our knowledge regarding the mechanisms of antibiotic resistance in *L. plantarum* and determine the need for comprehensive studies of the virulence evolution trajectories in lactic acid bacteria *in vivo* and *in vitro* to set probiotic virulence risk control strategies.

## MATERIALS AND METHODS

The strain *Lactiplantibacillus plantarum* 8p-a3 from the collection of microorganisms of the Molecular Genetics of Microorganisms Lab of the Institute of Fundamental Medicine and Biology of Kazan (Volga Region) Federal University (Kazan) isolated from the probiotic “Lactobacterin” (“Biomed,” Russia) was used in the work. The strain of *L. plantarum* 8p-a3-Clr-Amx, resistant to clinically significant concentrations of clarithromycin and amoxicillin-clavulanic acid (MIC values of 10 μg/mL and 20 μg/mL, respectively), was obtained as a result of sequential replating of *L. plantarum* 8p-a3 culture (MIC values of 0.5 μg/mL and 0.05 μg/mL, respectively) in MRS nutrient medium (BD Biosciences, USA) with an increasing concentration of antibiotics. The strains *L. plantarum* AG1 and AG10 from the collection of microorganisms of the Molecular Genetics of Microorganisms Lab of the Institute of Fundamental Medicine and Biology of Kazan (Volga Region) Federal University (Kazan) isolated from silage as described in (29) and the *L. plantarum* strain DMC-S1 isolated from the intestine of Drosophila melanogaster *Canton-S* line as described in (28) were used in the work to assess the virulence against *Drosophila*. The cultivation of lactic acid bacteria was carried out as described in ref. 69. To assess the resistance of *L. plantarum*, the MIC values of antimicrobial drugs were determined by microdilution according to the recommendations of EUCAST (26). In addition, the disk diffusion method was used to determine the sensitivity of lactic acid bacteria to antibiotics of different groups (27).

The growth parameters of *L. plantarum* were calculated as described in ref. 70. The generation time was calculated by the following formula: *g* = ln 2/*μ*, where *μ* is the specific growth rate (h^−1^). The specific growth rate was calculated by the formula *μ* = (ln *N*_t_ − ln *N*_0_)/(*t* − *t*_0_), where *μ* is the specific growth rate (h^−1^) and *N*_0_ and *N*_t_ are the optical density values of the culture at times *t*_0_ and *t*, respectively. The maximum specific growth rate was defined as the maximum value that takes *μ* between two dimensions. The lag time was defined as the time during which a strain of lactobacilli reaches the maximum specific growth rate.

To construct growth curves of *L. plantarum* strains, the optical density of cultures was measured using a spectrophotometer at a wavelength of 600 nm. For each strain, measurements were performed in three biological and three technical repetitions. The graphs were plotted based on the mean values, and the standard deviation was determined. The data were compared using Tukey’s multiple-comparison test using an ordinary one-way analysis of variance (ANOVA). To determine CFU values in *L. plantarum* cultures at different stages of growth (lag, middle, second half of the log phase, and stationary phase), the drop plate method was used (71). All strains were compared with each other in pairs using the Kruskal-Wallis criterion. The differences were considered significant at a *P* value of <0.05. The axenicity of the culture was tested using transmission electron microscopy (72) and PCR using universal and specific probes complementary to the 16S rRNA and esterase genes (locus E3U93_04390), respectively.

The genomes of *L. plantarum* strains (8p-a3, 8p-a3-Clr-Amx) were sequenced on the MiSeq platform (Illumina, USA). For the analysis of nucleotide sequences, the Sequencing Analysis 5.3.1 program (Applied Biosystems, USA) was used as well as the NCBI database. Bowtie2 (http://bowtie-bio.sourceforge.net/bowtie2/index.shtml) was used for the alignment of nucleotide sequences, and SAMtools (http://samtools.sourceforge.net/mpileup.shtml) and SnpEff (http://snpeff.sourceforge.net/SnpEff.html) were used for the search and annotation of single nucleotide polymorphisms (SNPs) accordingly. Comparison of nucleotide sequences was performed using the BLAST algorithm.

The search for resistance genes was performed using the hidden Markov model (HMM) algorithm and the Resfams database. To search for antibiotic resistance genes, the obtained assemblies were mapped to the database of antibiotic resistance genes CARD (https://card.mcmaster.ca/home). The search for mobile genetic elements was performed using VRprofile 2.0 (https://tool-mml.sjtu.edu.cn/STEP/STEP_VR.html). VFDB databases and VRprofile 2.0 were used to search for proteins associated with bacterial virulence (http://www.mgc.ac.cn/VFs/main.htm).

The virulence of *L. plantarum* strains was evaluated in relation to Drosophila melanogaster of the *Canton-S* line, the profile of the intestinal microbiota of which is presented by us in the SRA database (accession number PRJNA751047). Flies were cultured on a standard sugar-yeast nutrient medium and kept at 25°C in a thermostat and 12 h in lighting mode. Infection of flies with *L. plantarum* 8p-a3 and *L. plantarum* 8p-a3-Clr-Amx strains was performed through a nutrient substrate according to ref. 60. To do this, synchronous embryo clutches were obtained, which were transferred to the surface of the nutrient medium with the addition of 100 μL of lactic acid bacterium cells washed in phosphate-buffered saline (PBS; CFU of 10^6^) (73). Flies grown on a medium that did not contain *L. plantarum* were used as a control. Control of *Drosophila* infection with *Lactobacillus* strains was performed using serial dilutions of homogenate from the intestine by the drop plate method (71) with subsequent seeding on MRS medium as well as PCR. Amplification of *L. plantarum* nucleotide sequences was performed using PCR with universal primers 341F 5’-CCTACGGGAGGCAGCAG-3’ and 926R 5’-CCGTCAATTCCTTTGAGTTT-3’ and with specific primers Lp1F 5’-GCACTGGCTAATAACAGTC-3’, Lp1R 5’-CATCGCTTACTGACTGAGT-3’, Lp2F 5’-CGTTTCGGATAGTGCCCTT-3’, Lp2R 5’-ACCGATCCCCGTCACTTTA-3’, Lp3F 5’-TGAGAAGGTTGGTAAGCC-3’, and Lp3R 5’-TTCACGGCTATCTGAGGT-3’ in the following modes: for 341F-926R 95°C for 3 min (95°C for 15 s; 54°C for 15 s; 72°C for 10 s; 18 cycles), for Lp1 95°C for 3 min (95°C for 10 s; 45°C for 5 s; 72°C for 15 s; 18 cycles), for Lp2 95°C for 3 min (95°C for 5 s; 55°C for 5 s; 72°C for 10 s; 18 cycles), and for Lp3 95°C for 3 min (95°C for 5 s; 45°C for 5 s; 72°C for 5 s; 18 cycles). To increase the sensitivity and specificity of the reaction, amplification products obtained using Lp1 primers were used as a matrix for nested PCR with Lp2 primers under the same temperature and time conditions.

To assess the virulence of *L. plantarum* strains, standard indicators of reproduction and viability of individuals were used. To do this, the number of eggs laid, surviving embryos, and the egg production index were determined in infected and uninfected fruit flies (74–76).

To assess DNA damage in the enterocytes of flies, an alkaline variant of the DNA comet assay was used, which allows for the determination of single-strand DNA breaks in cells (77). A fluorescence microscope was used to visualize and rank the DNA comets (Carl Zeiss Axio Imager M2, Germany). To assess the damage to the intestinal tissue of infected fruit flies, conventional staining techniques were used, including trypan blue (78), propidium iodide (79), Hoechst (80), and 4′,6-diamidino-2-pheylindole (DAPI) (81). A fluorescence microscope was used for visualization (Carl Zeiss Axio Imager M2, Germany). The resulting photos were processed using the ImageJ program.

The survival rate of flies was assessed according to ref. 82. Survival curves are displayed as Kaplan-Meier graphs constructed using GraphPad Prism version 6.0.

Statistical data processing was performed using the software Statistica 12.0 using one-factor analysis of variance (one-way ANOVA) using a Bonferroni *post hoc* test and GraphPad Prism version 6.0 for Windows (GraphPad Software). Experiments were performed in three repetitions. For each indicator, the arithmetic mean, its error, and standard deviation were calculated. For the growth parameters, statistically significant differences were determined using Tukey’s multiple-comparison test using an ordinary one-way ANOVA (*P* < 0.05). Statistical analysis of data on the survival of flies was performed using the log-rank (Mantel-Cox) test. The relative mortality risk (RR) was assessed using the Cox proportional hazards model using SPSS Statistics version 18.0. The differences were considered significant at a *P* value of <0.05.

### Data availability.

The whole-genome sequences of *L. plantarum* 8p-a3 and *L. plantarum* 8p-a3-Clr-Amx were submitted to the GenBank database under accession number PRJNA528387, and the profile of the D. melanogaster intestinal microbiota was submitted to the SRA database under accession number PRJNA751047.
