# Supplementary material for: Development of Resistance to Clarithromycin and Amoxicillin-Clavulanic Acid in Lactiplantibacillus plantarum In Vitro Is Followed by Genomic Rearrangements and Evolution of Virulence
Source: Microbiol Spectr. 2022 May 17;10(3):e02360-21. doi: 10.1128/spectrum.02360-21 (PMC9241834; doi:10.1128/spectrum.02360-21)
Supplement: SUPPLEMENTAL FILE 1 — Supplemental material. Download spectrum.02360-21-s0001.pdf, PDF file, 1.3 MB [file spectrum.02360-21-s0001.pdf]

Appendix

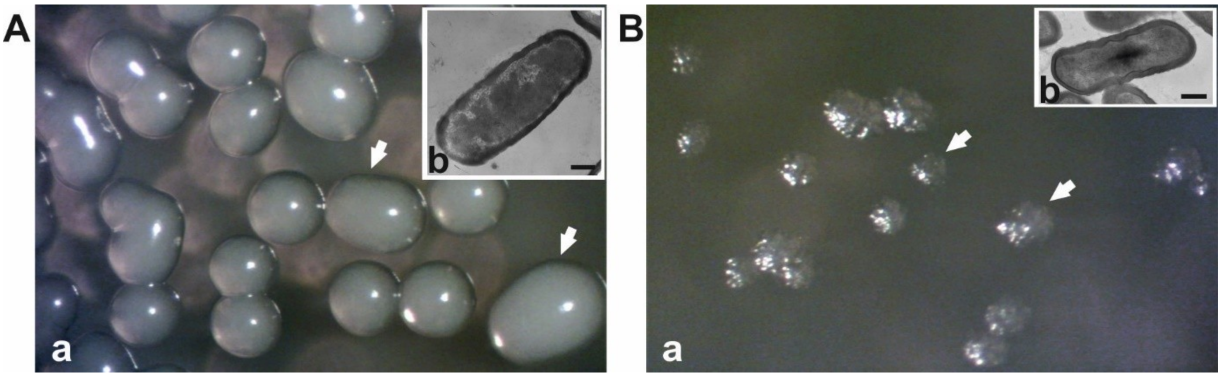

Figure S1. Morphology of *L. plantarum* colonies and cells 8p-a3 (A) and 8p-a3-Clr-Amx (B).

a - Micrographs of *L. plantarum* colonies on agarized MRS. Obtained at the same magnification using an optical microscope «OPTIKA» (digital camera - eyepiece 2 MPixels Levenhuk M200 BASE). The arrows indicate individual colonies.

b - TEM of *L. plantarum* cells. The scale segment (bar) corresponds to 200 nm. Cells for microscopy were grown in MRS medium at 37°C and collected in exponential growth phase.

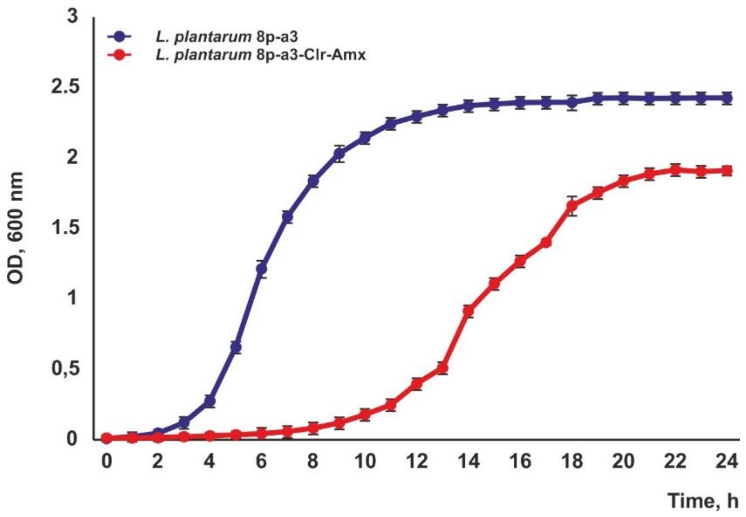

Figure S2. Growth curves of *L. plantarum* 8p-a3 and *L. plantarum* 8p-a3-Clr-Amx on the MRS nutrient medium. The data is presented in the form of averages and standard deviation. For each strain, measurements were carried out in three biological and three technical repetitions.

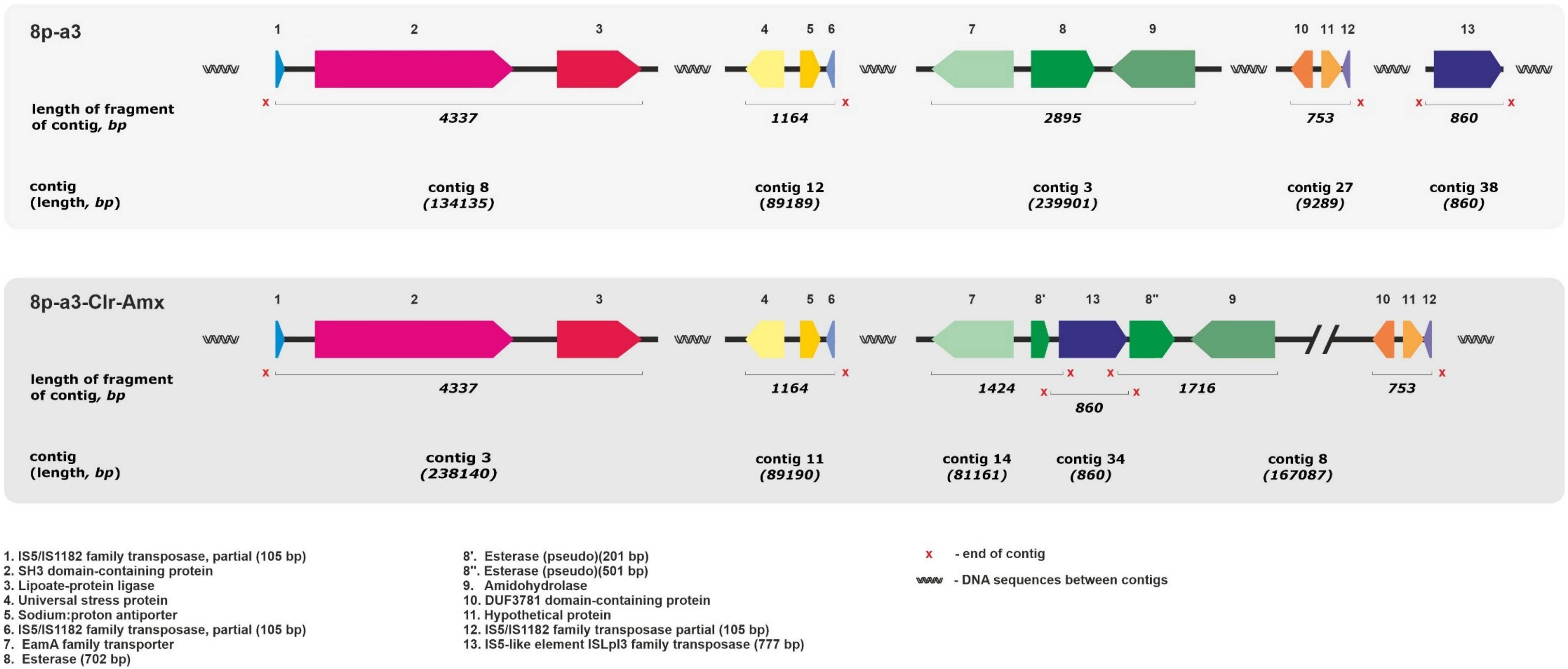

Figure S3. Features of the location of transposase genes (including partially sequenced ones) on the contigs of *L. plantarum* strains 8p-a3 and 8p-a3-Clr-Amx, which could move to the esterase gene when the bacterium adapts to clarithromycin and amoxicillin-clavulanic acid.

To determine the possible order of contigs, the genome of the reference strain *L. plantarum* 8P-A3 (GenBank NZ_CP046726.1) was used.

In strain 8p-a3-Clr-Amx, a transposase (MBW2744515.1) was embedded in the gene encoding the esterase (contig JAHWFM010000008.1, locus KXC18_09050), part (105 nucleotides) of the sequence of which was sequenced. Fragments of the esterase gene were detected in two contigs of strain 8p-a3-Clr-Amx - JAHWFM010000008.1 (locus KXC18_09050, 501 nucleotides) and JAHWFM010000014.1 (locus KXC18_12345, 201 nucleotides).

The localization of transposase inside the esterase gene in strain 8p-a3-Clr-Amx was confirmed by targeted sequencing of the site using F. Sanger method. In strain 8p-a3, the gene encoding the corresponding esterase (TFE51650.1) has a length of 702 nucleotides.

The identical sequence of the transposase part was sequenced at 4 loci in 4 different contigs of strain 8p-a3 (SOQA01000008.1, SOQA01000012.1, SOQA01000027.1 and SOQA01000038.1) and at 5 loci in 4 different contigs of strain 8p-a3-Clr-Amx (JAHWFM010000003.1, JAHWFM010000011.1, JAHWFM010000008.1 and JAHWFM010000034.1). Comparisons of nucleotide sequences were carried out using the BLAST algorithm.

Transposase embedding into the homologous esterase gene was not detected in any sequenced *L. plantarum* spps.

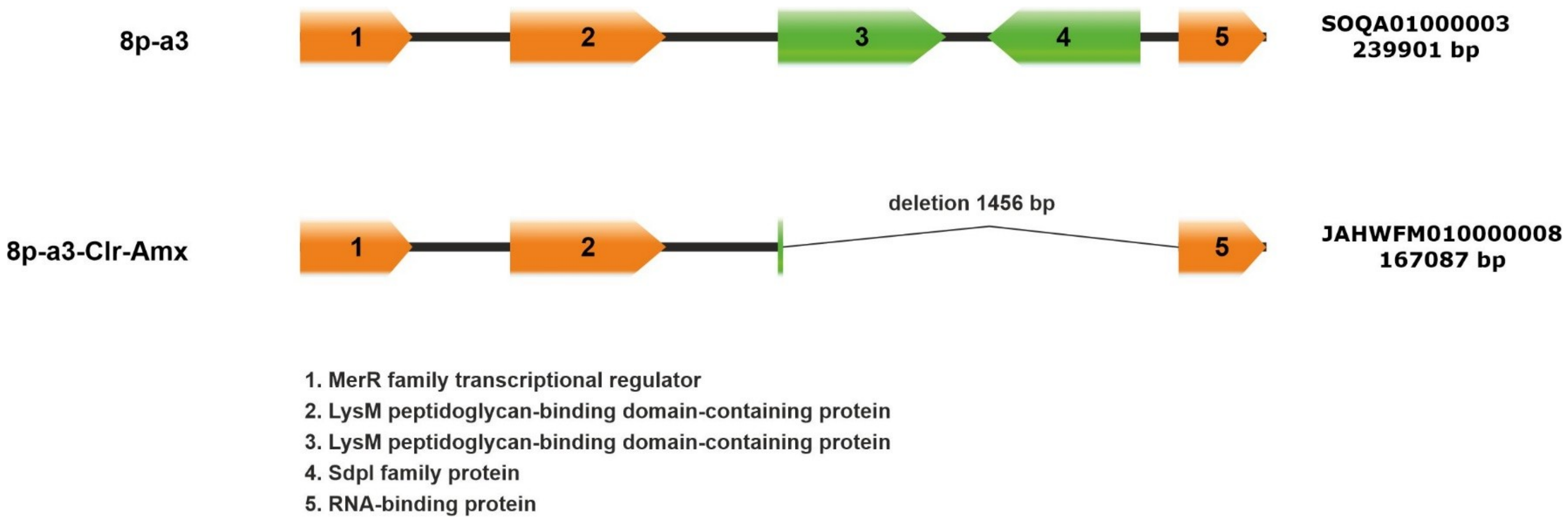

Figure S4. Scheme of alignment of nucleotide sequences of *L. plantarum* strains 8p-a3 and 8p-a3-Clr-Amx with detected fragment deletions.

The strain 8p-a3-Clr-Amx revealed deletion of 1456 nucleotides in the contig JAHWFM010000008.1. Strain 8p-a3 has two genes in the corresponding position (contig SOQA01000003.1) that encode a protein containing the peptidoglycan-binding domain LysM (TFE51695.1) and SdpI family protein (TFE51696.1). The deleted genes in strain 8p-a3-Clr-Amx have not been sequenced in other contigs of the bacterium. Previously, deletion of these two genes was not detected in other strains of *L. plantarum* and, thus, is strain-specific.

Comparisons of nucleotide sequences of two *L. plantarum* strains were carried out using the BLAST algorithm.

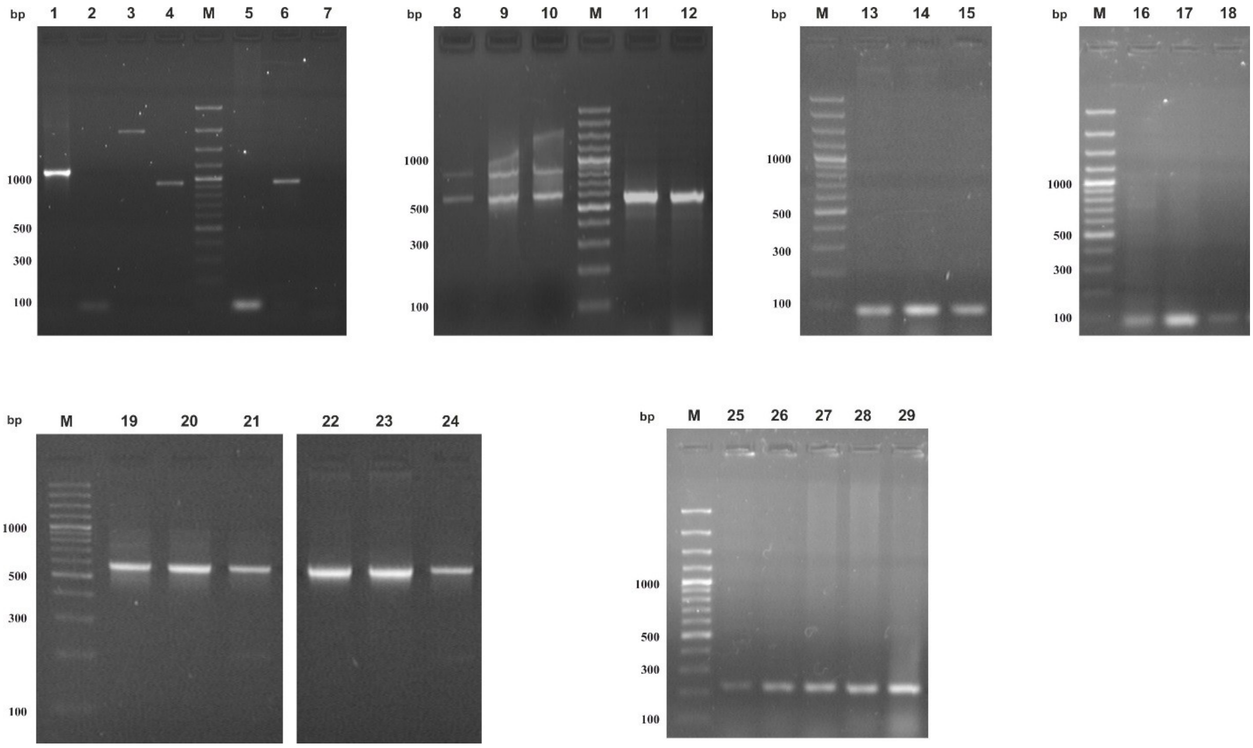

Figure S5. Electrophoregrams of amplification products obtained in PCR using DNA of *L. plantarum* 8p-a3 (1, 2, 11, 25), 8p-a3-Clr-Amx (3, 4, 12, 26), AG1 (13, 19, 27), AG10 (14, 20, 28) and DMC-S1 (15, 21, 29), as well as total DNA from *D. melanogaster*, not infected (7, 10), as well as infected with *L. plantarum* 8p-a3 (5, 8), *L. plantarum* 8p-a3-Clr-Amx (6, 9), AG1 (16, 22), AG10 (17, 23) and DMC-S1 (18, 24) and primers Lp1 (1, 3), Lp2 (2, 4-7, 13-18), Lp3 (25-29) and 341F-926R (8-12, 19-24). M – marker (DNA Ladder). Amplicons 1, 2, 3 and 4 were sequenced by Sanger's method.

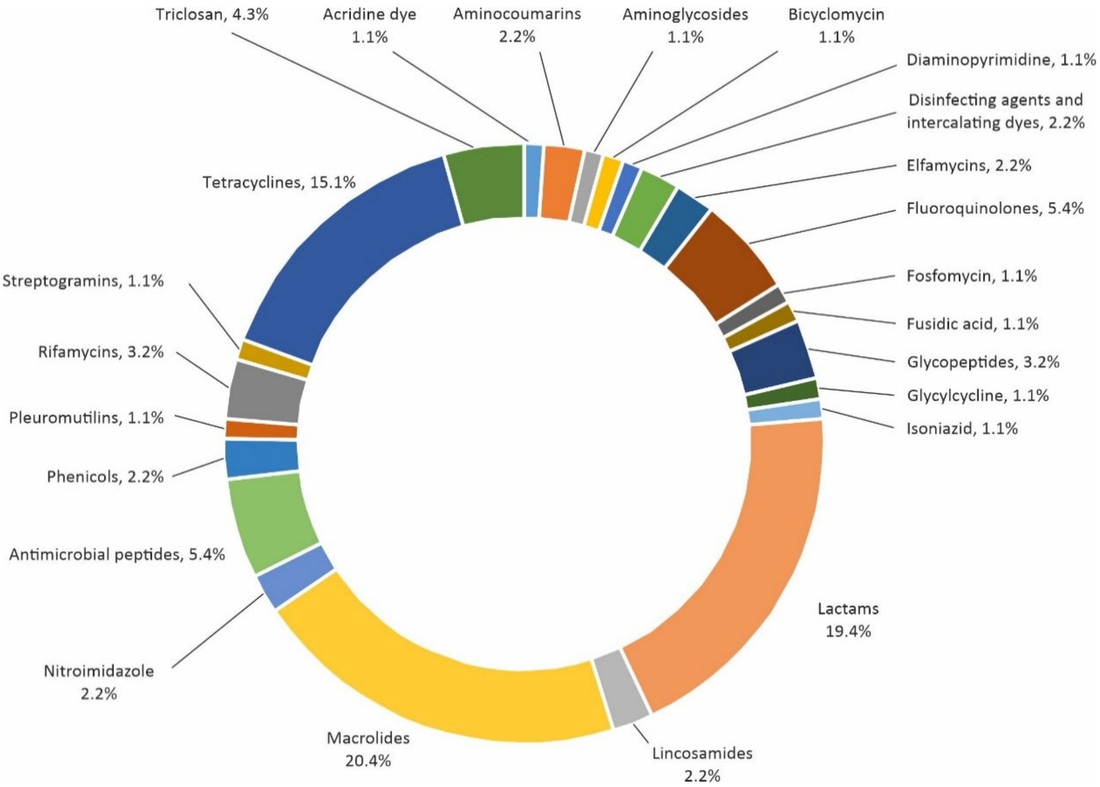

Figure S6. Diagram of the representation of *L. plantarum* 8p-a3 genes associated with resistance to antibacterial compounds of different classes (according to CARD).

The percentage (%) of each class of genes is indicated. A complete list of resistance-associated genes and encoded proteins is given in Table S2.

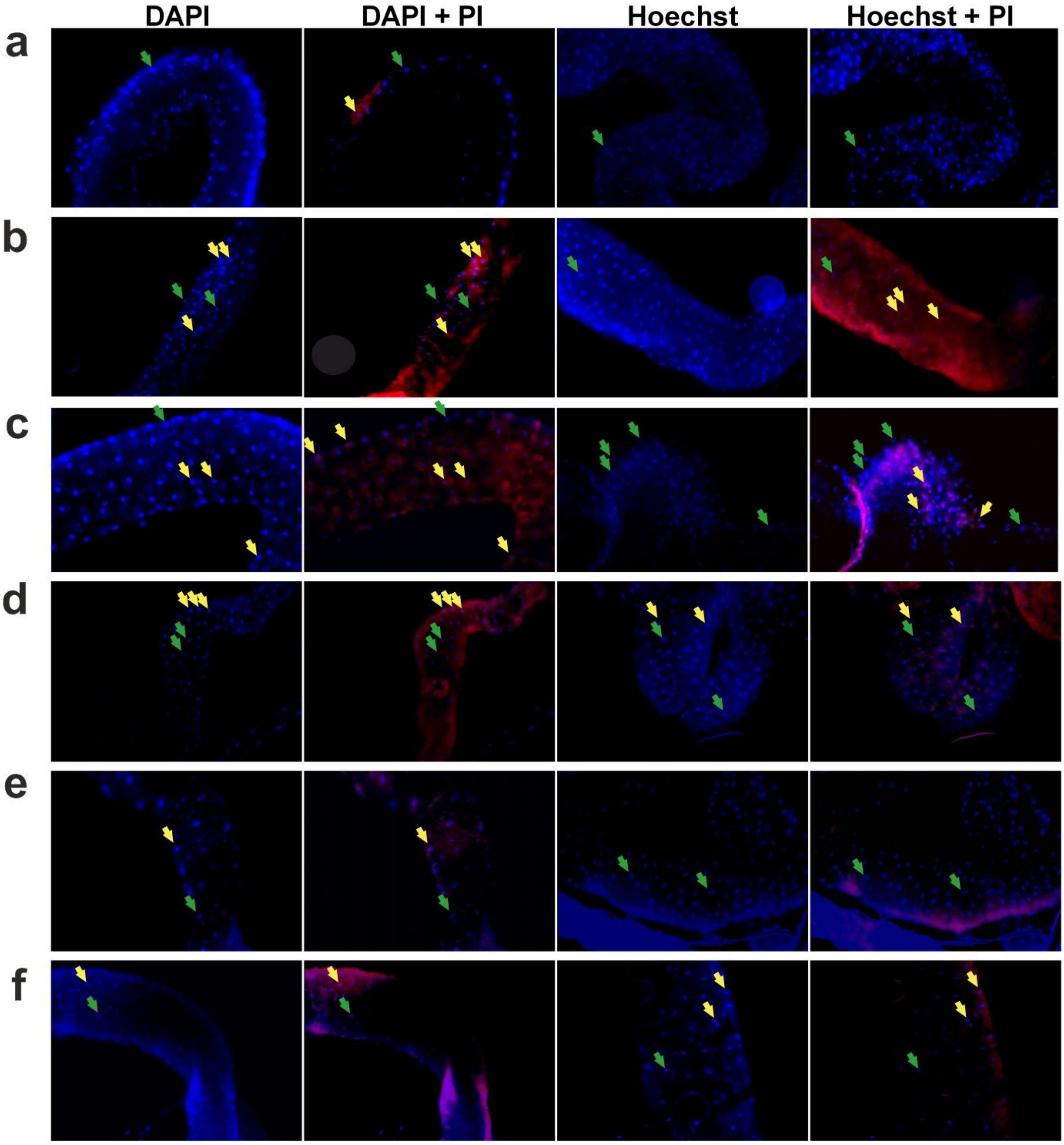

Figure S7. The effect of *L. plantarum* on the enterocytes of *D. melanogaster*. Micrographs of fly gut preparations stained with DAPI, propidium iodide (PI) and Hoechst dyes (magnification 20x).

a – intestinal preparations of flies not infected with *L. plantarum*, b, c, d, e and f – intestinal preparations of flies infected with *L. plantarum* 8p-a3, 8p-a3-Clr-Amx, AG1, AG10 and DMC-S1 respectively.

Morphological changes in the nuclei of intestinal cells were determined using a fluorescent microscope by staining the drug with dyes DAPI (blue glow; dye binds to nucleic acids, penetrating through the cytoplasmic membrane of both viable and dead cells), Hoechst (blue

glow; dye binds to nucleic acids, penetrating through the cytoplasmic membrane of a viable cell), PI (red glow; dye binds to fragmented/condensed nuclei, penetrating through the damaged cytoplasmic membrane of the cell). Double staining of DAPI + PI and Hoechst + PI was used, allowing differential assessment of cell viability. In the presented intestinal segments of intact and infected flies with *L. plantarum* strains, green arrows indicate viable cells with an intact nucleus (the nuclei are colored blue), yellow arrows indicate necrotic/apoptotic cells with a fragmented nucleus (the nuclei are colored pink-red). The fluorescence intensity was determined using ImageJ software. The data obtained were compared using One-way ANOVA with Bonferroni post-hoc test. The differences were considered significant at $p < 0.05$.

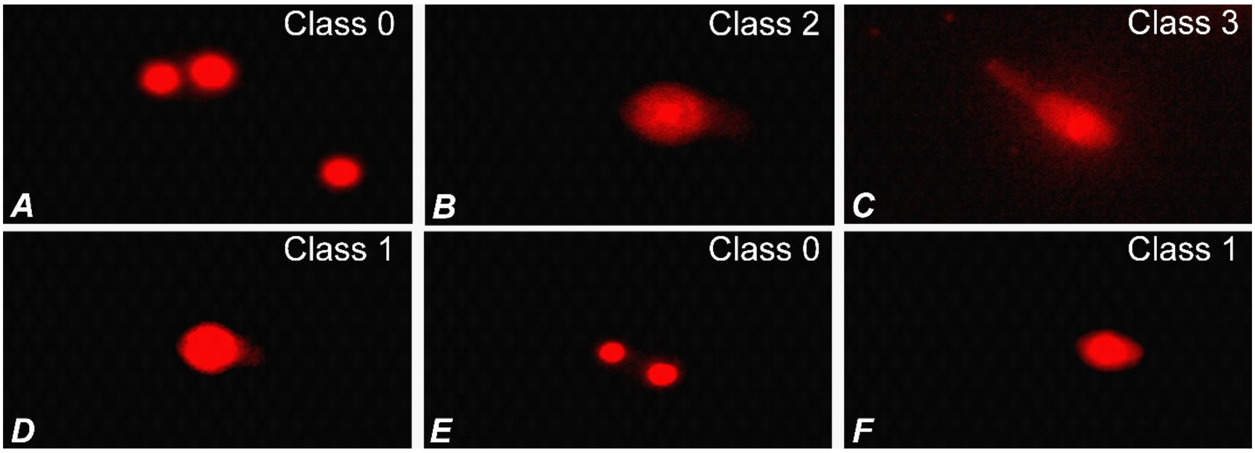

Figure S8. The main types of DNA-comets observed in specimens from the enterocytes of the intestine of *D. melanogaster*, not infected (A) and infected with *L. plantarum* 8p-a3 (B), 8p-a3-Clr-Amx (C), AG1 (D), AG10 (E) and DMC-S1 (F).

Class 0 - nucleus of cells containing non-fragmented DNA, class 1 – tail up to 1.5 times the diameter of the comet nucleus, class 2 and class 3 – around the nucleus is visualized halo, associated with the violation of the integrity of DNA. An increase in the length of the comet's tail develops in parallel with a decrease in the content of nuclear DNA. The DNA damage index was calculated using the formula $(0 \times n_0 + 1 \times n_1 + 2 \times n_2 + 3 \times n_3 + 4 \times n_4) / \Sigma$, where n_0-n_4 is the number of DNA comets of each type, Σ is the sum of the analyzed DNA comets.

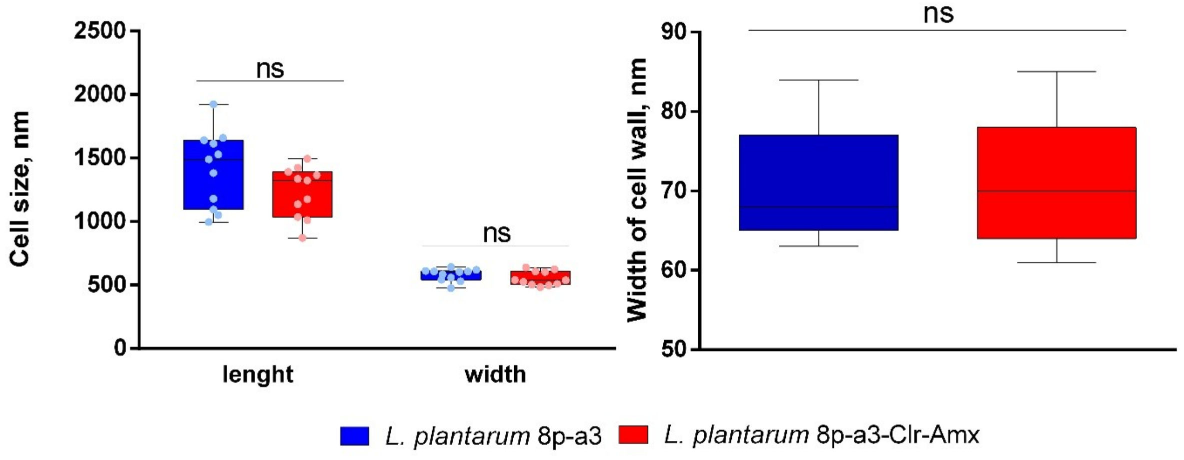

Figure S9. Cell sizes of *L. plantarum* 8p-a3 and 8p-a3-Clr-Amx.

The box-and-whisker plots show the average, 75% quartiles and extremes values. ns – difference is not significant ($p > 0.05$; One-way ANOVA).

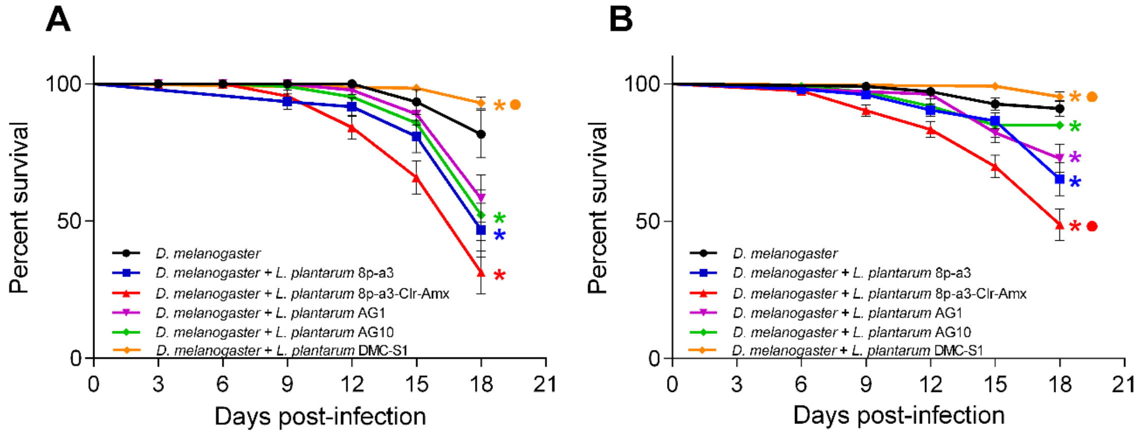

Figure S10. Survival curves of females (A) and males (B) after infection with *L. plantarum* strains of different origin. The number of females and males in each group was at least 150 individuals. The significance of the differences between the survival curves was assessed using the log-rank (Mantel-Cox) test on day 18 after the start of infection (* $p < 0.05$ compared with the control uninfected group; • $p < 0.05$ compared with infection with the *L. plantarum* strain 8p-a3).

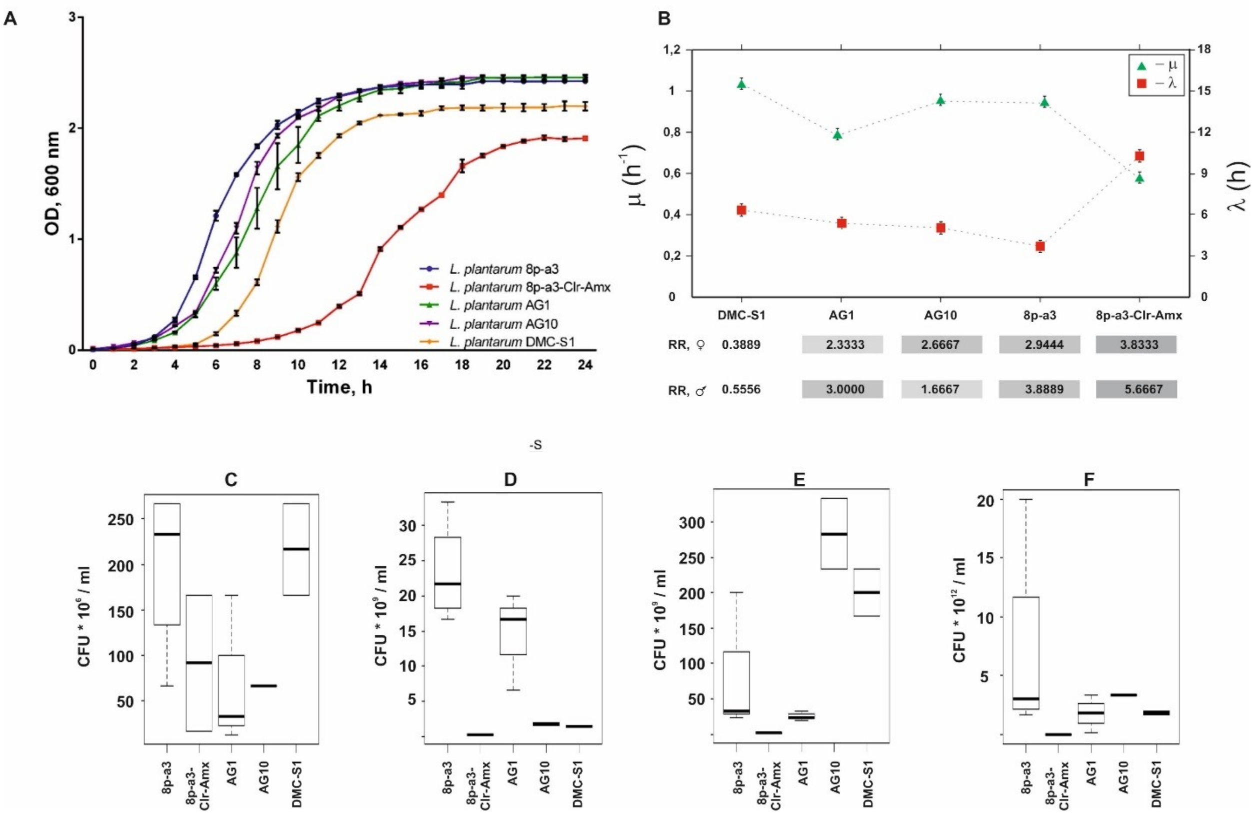

Figure S11. Growth curves (A), maximum specific growth rate, lag time (B) and colony forming unit (C-F) of *L. plantarum* 8p-a3, AG1, AG10, DMC-S1 and 8p-a3-Clr-Amx on the MRS nutrient medium. (A) The data is presented in the form of averages and standard deviation. (C-F) The data are presented as medians of CFU values in *L. plantarum* cultures at the beginning (C), middle (D), second half (E) of the log phase and stationary (F) growth phase. All strains were compared with each other in pairs using the Kruskal-Wallis criterion. For each strain, measurements were carried out in three biological repetitions. μ_{max} – maximum specific growth rate, λ - lag time, RR – relative risk of mortality for males (♂), females (♀) of *D. melanogaster*.

Table S1. Indicators of the specific growth rate and generation time of *L. plantarum* 8p-a3 and 8p-a3-Clr-Amx when cultivating bacteria on the MRS nutrient medium

**#**	**Strain**	**μ, h^{-1}**	**Generation time, h**
1	*L. plantarum* 8p-a3	0.745 ± 0.035	0.932 ± 0.044
2	*L. plantarum* 8p-a3-Clr-Amx	0.387 ± 0.01*	1.792 ± 0.046**

The data is presented in the form of averages and standard deviation.

*$p = 0.0001$; ** $p < 0.0001$.

Table S2. *L. plantarum* 8p-a3 and *L. plantarum* 8p-a3-Clr-Amx proteins associated with antibiotic resistance (based on CARD)

**№**	**Protein of *L. plantarum***	**Protein ID**	**Homologous protein in the CARD database**	**Ident %**	**E-value**	**Bit score**	**Drug Class**
1	AraC family transcriptional regulator	TFE51995.1/MBW2743266.1	gadX|Escherichia_coli_str._K-12_substr._W3110	23.53	8e-08	48.9	penam, macrolide antibiotic, fluoroquinolone antibiotic
2	ATP-binding cassette domain-containing protein	TFE51142.1/MBW2744025.1	tetA(48)|Paenibacillus_sp._LC231	50.32	1e-96	290	tetracycline antibiotic
3	Bcr/CflA family efflux MFS transporter	TFE51951.1/MBW2743221.1	bcr-1|Pseudomonas_aeruginosa	32.08	5e-31	119	bicyclomycin
[4]	bifunctional lysylphosphatidylglycerol flippase/synthetase MprF	TFE52434.1/pseudo	Listeria_monocytogenes_mprF| Listeria_monocytogenes_EGD-e	46.19	0	781	peptide antibiotic
5	chloramphenicol acetyltransferase CAT	TFE50887.1/MBW2744184.1	catQ|Clostridium_perfringens	31.68	5e-36	125	phenicol antibiotic
6	class A beta-lactamase-related serine hydrolase	TFE51055.1/MBW2744112.1	Escherichia coli ampH beta-lactamase	21	1.8e-11	61	penam, cephalosporin
7	class A beta-lactamase-related serine hydrolase	TFE49101.1/MBW2745283.1	Escherichia coli ampH beta-lactamase	25	1.1e-13	68	penam, cephalosporin
8	class A beta-lactamase-related serine hydrolase	TFE48225.1/MBW2745688.1	Yrc-1|Yersinia ruckeri	23	6.9e-11	61	penam, cephalosporin
9	dihydrofolate reductase	TFE51175.1/MBW2743992.1	dfrE|Enterococcus_faecalis_EnGen0074	43.03	4e-39	130	diaminopyrimidine antibiotic
10	DNA-directed RNA polymerase subunit beta	TFE52697.1/MBW2743083.1	Nocardia_rifampin_resistant_beta-subunit_of_RNA_polymerase_(rpoB2)| Nocardia_farcinica_IFM_10152	61.54	0	1006	rifamycin antibiotic
11	elongation factor G-binding protein	TFE50591.1/MBW2744390.1	FusF|Staphylococcus_cohnii	40.95	7e-51	164	fusidic acid
12	glycosyltransferase	TFE49944.1/MBW2744838.1	pmrF|Escherichia_coli_str._K-12_substr._MG1655	23.24	1e-06	46.2	pmr phosphoethanolamine transferase
13	LysR family transcriptional regulator	TFE48214.1/MBW2745699.1	NmcR|Enterobacter_cloacae	35	1e-08	51.2	penam, cephalosporin, carbapenem, cephamycin
14	potassium transporter	TFE52610.1/MBW2742996.1	rosB|Yersinia_enterocolitica_(type_O:8)	25.77	8e-06	45.1	peptide antibiotic
15	response regulator transcription factor	TFE48498.1/MBW2745548.1	vanRF|Paenibacillus_popilliae_ATCC_14706	50.44	3e-73	222	glycopeptide antibiotic
16	serine hydrolase	TFE49760.1/MBW2743699.1	Escherichia_coli_ampC1_beta-lactamase| Escherichia_coli_ETEC_H10407	27.3	4e-23	95.5	cephalosporin, penam
17	serine hydrolase	TFE48037.1/MBW2745714.1	Escherichia_coli_ampC1_beta-lactamase| Escherichia_coli_ETEC_H10407	31.21	1e-33	126	cephalosporin, penam
18	serine hydrolase	TFE51937.1/MBW2743208.1	blaF| Mycolicibacterium fortuitum	30	3.8e-05	40	penam
19	serine hydrolase	TFE48037.1/MBW2745714.1	Escherichia coli ampC1 beta-lactamase	31	1.5e-33	126	cephalosporin, penam

20	tetracycline resistance MFS efflux pump	TFE52502.1/MBW2742886.1	emeA|Enterococcus_faecalis_ATCC_29212	41.3	3e-87	270	disinfecting agents and intercalating dyes, acridine dye
21	undecaprenyl-diphosphate phosphatase	TFE51470.1/MBW2743799.1	bacA|Escherichia_coli_str._K-12_substr._MG1655	41.03	7e-54	176	peptide antibiotic
22	VanZ family protein	TFE50312.1/MBW2743577.1	vanZF|Paenibacillus_popilliae_ATCC_14706	30.23	7e-07	46.2	glycopeptide antibiotic
23	ABC transporter ATP-binding protein	TFE51715.1/MBW2744578.1	lmrD|Lactococcus_lactis	56.18	0	624	lincosamide antibiotic
24	ABC transporter ATP-binding protein	TFE51716.1/MBW2744579.1	patA|Streptococcus_pneumoniae_TIGR4	49.82	0	591	fluoroquinolone antibiotic
25	ABC transporter ATP-binding protein	TFE51173.1/MBW2743994.1	TaeA|Paenibacillus_sp._LC231	43.68	0	557	pleuromutilin antibiotic
26	multidrug efflux SMR transporter	TFE48946.1/MBW2745391.1	abeS|Acinetobacter_baumannii_AB307-0294	32.71	1e-13	60.1	macrolide antibiotic, aminocoumarin antibiotic
27	ABC transporter ATP-binding protein	TFE49910.1/MBW2744872.1	bcrA|Bacillus_licheniformis	28.91	1e-27	105	peptide antibiotic
28	MFS transporter	TFE51593.1/MBW2745190.1	efpA|Mycobacterium_tuberculosis_H37Rv	30.42	2e-39	146	rifamycin antibiotic, isoniazid
29	ABC transporter ATP-binding protein	TFE49766.1/MBW2743693.1	efrA|Enterococcus_faecalis	41.3	2e-147	437	macrolide antibiotic, rifamycin antibiotic, fluoroquinolone antibiotic
30	MFS transporter	TFE50805.1/MBW2744266.1	facT|Streptomyces_sp._WAC5292	27.22	9e-36	137	elfamycin antibiotic
31	MFS transporter	TFE52092.1/MBW2743364.1	facT|Streptomyces_sp._WAC5292	28.51	1e-35	135	elfamycin antibiotic
32	MFS transporter	TFE49604.1/MBW2744955.1	lmrP|Streptococcus_pyogenes_MGAS9429	23.47	1e-08	52.4	macrolide antibiotic, lincosamide antibiotic, streptogramin antibiotic, tetracycline antibiotic
33	ATP-binding cassette domain-containing protein	TFE48270.1/MBW2744681.1	macB|Neisseria_gonorrhoeae	28.53	1e-65	227	macrolide antibiotic
34	ABC transporter ATP-binding protein	TFE48529.1/MBW2745579.1	macB|Neisseria_gonorrhoeae	39.11	6e-44	154	macrolide antibiotic
35	ATP-binding cassette domain-containing protein	TFE50360.1/MBW2743626.1	macB|Neisseria_gonorrhoeae	28.86	6e-75	253	macrolide antibiotic
36	ATP-binding cassette domain-containing protein	TFE51682.1/MBW2744547.1	macB|Neisseria_gonorrhoeae	29.18	5e-75	253	macrolide antibiotic
37	ABC transporter ATP-binding protein	TFE52074.1/MBW2743346.1	macB|Neisseria_gonorrhoeae	30.4	7e-31	117	macrolide antibiotic
38	ABC transporter permease	TFE52075.1/MBW2743347.1	macB|Neisseria_gonorrhoeae	25.34	6e-09	54.3	macrolide antibiotic
39	MFS transporter	TFE50544.1/MBW2744435.1	mdtG| Escherichia_coli_O139:H28_str._E24377A	24.7	2e-08	52	fosfomycin
40	MFS transporter	TFE52776.1/MBW2742939.1	mefB|Escherichia_coli	22.79	6e-08	49.3	macrolide antibiotic

41	MATE family efflux transporter	TFE52238.1/MBW2743516.1	mepA|Staphylococcus_aureus	30.66	1e-59	201	tetracycline antibiotic, glycylcycline
42	TetR/AcrR family transcriptional regulator	TFE50586.1/MBW2744395.1	mexL|Pseudomonas_aeruginosa_PAO1	35.9	4e-08	47.8	tetracycline antibiotic, macrolide antibiotic, triclosan
43	TetR/AcrR family transcriptional regulator	TFE51778.1/MBW2744641.1	mexL|Pseudomonas_aeruginosa_PAO1	36.49	8e-07	44.7	tetracycline antibiotic, macrolide antibiotic, triclosan
44	TetR/AcrR family transcriptional regulator	TFE52185.1/MBW2743457.1	mexL|Pseudomonas_aeruginosa_PAO1	39.66	4e-07	45.8	tetracycline antibiotic, macrolide antibiotic, triclosan
45	TetR/AcrR family transcriptional regulator	TFE52696.1/MBW2743082.1	mexL|Pseudomonas_aeruginosa_PAO1	42.11	1e-10	55.1	tetracycline antibiotic, macrolide antibiotic, triclosan
46	ABC transporter ATP-binding protein	TFE50362.1/MBW2743628.1	msbA|Escherichia_coli_str._K-12_substr._MG1655	29.72	1e-80	264	nitroimidazole antibiotic
47	ABC transporter ATP-binding protein	TFE51655.1/MBW2744520.1	msbA|Escherichia_coli_str._K-12_substr._MG1655	31.27	9e-80	262	nitroimidazole antibiotic
48	ABC transporter ATP-binding protein	TFE50498.1/MBW2744482.1	novA|Streptomyces_niveus	32.81	1e-112	350	aminocoumarin antibiotic
49	ABC transporter ATP-binding protein	TFE49658.1/MBW2744902.1	oleC|Streptomyces_antibioticus	28.97	6e-18	79	macrolide antibiotic
50	ABC transporter ATP-binding protein	TFE50281.1/MBW2743546.1	oleC|Streptomyces_antibioticus	25.11	1e-14	68.2	macrolide antibiotic
51	ABC transporter ATP-binding protein	TFE52046.1/MBW2743318.1	oleC|Streptomyces_antibioticus	26.01	4e-16	73.6	macrolide antibiotic
52	ABC transporter ATP-binding protein	TFE52187.1/MBW2743459.1	oleC|Streptomyces_antibioticus	30.07	9e-37	132	macrolide antibiotic
53	ABC transporter ATP-binding protein	TFE50497.1/MBW2744483.1	patA|Streptococcus_pneumoniae_TIGR4	32.01	2e-88	284	fluoroquinolone antibiotic
54	ABC transporter ATP-binding protein	TFE49765.1/MBW2743694.1	patB|Streptococcus_pneumoniae_TIGR4	38.17	2e-122	375	fluoroquinolone antibiotic
55	multidrug efflux SMR transporter	TFE48895.1/MBW2745392.1	qacH|Vibrio_cholerae	34.41	3e-15	63.9	disinfecting agents and intercalating dyes
56	MFS transporter	TFE50105.1/MBW2744790.1	tcr3|Kitasatospora_aureofaciens	28.91	3e-49	175	tetracycline antibiotic
57	ABC transporter ATP-binding protein	TFE48368.1/MBW2745650.1	tetA(48)|Paenibacillus_sp._LC231	33.64	6e-40	140	tetracycline antibiotic
58	ABC transporter ATP-binding protein	TFE50127.1/MBW2744766.1	tetA(48)|Paenibacillus_sp._LC231	33.66	1e-49	167	tetracycline antibiotic
59	ABC transporter ATP-binding protein	TFE50772.1/MBW2744299.1	tetA(48)|Paenibacillus_sp._LC231	29.82	1e-37	133	tetracycline antibiotic
60	ABC transporter ATP-binding protein	TFE51104.1/MBW2744063.1	tetA(48)|Paenibacillus_sp._LC231	31.78	4e-30	114	tetracycline antibiotic
61	ABC transporter ATP-binding protein	TFE51603.1/MBW2745181.1	tetB(60)|uncultured_bacterium	38.73	1e-117	362	tetracycline antibiotic
62	multidrug efflux SMR transporter	TFE52606.1/MBW2742992.1	ykkD|	29.81	1e-13	60.1	phenicol antibiotic, tetracycline antibiotic,

			Bacillus_subtilis_subsp._subtilis_str._168				aminoglycoside antibiotic
63	D-alanine--D-alanine ligase	TFE49728.1/MBW2743732.1	D-Ala-D-Ala ligase|Enterococcus gallinarum	36	7e-76	239	glycopeptide antibiotic

[ ] - a mutation was detected in the corresponding gene in the *L. plantarum* strain 8p-a3-Clr-Amx.

Table S3. Genes of *L. plantarum* 8p-a3 and *L. plantarum* 8p-a3-Clr-Amx in which IS elements are predicted (according to the VRprofile database).

№	Locus of *L. plantarum* 8p-a3[1]	Locus of *L. plantarum* 8p-a3-Clr-Amx[1]	Protein[2]	Hit	Ha-value
1	SOQA01000012.1, 1-60	JAHWFM010000011.1, 89130-89189		ISLpl3_PEP3	1.000
2	SOQA01000020.1, 59148-59207	JAHWFM010000020.1, 59148-59207		ISLpl3_PEP3	1.000
3	E3U93_09125/TFE50261.1	KXC18_03925/MBW2743525.1	IS5/IS1182 family transposase (partial)	ISLpl3_PEP3	1.000
4	E3U93_11885/TFE49662.1	KXC18_11085/MBW2744898.1	IS5/IS1182 family transposase (partial)	ISLpl3_PEP3	1.000
5	E3U93_14600/TFE48474.1	KXC18_14445/MBW2745524.1	IS5/IS1182 family transposase (partial)	ISStma16_PEP2	0.730
6	E3U93_10920/TFE49705.1	KXC18_05135/MBW2743755.1	ISL3 family transposase (partial)	ISP1_PEP	0.808
7	SOQA01000020.1, 45207-45371	JAHWFM010000020.1, 45207-45371		ISLsa2_PEP3	0.685
8	E3U93_01440/TFE52658.1	KXC18_01405/MBW2743043.1	helix-turn-helix domain-containing protein	ISSau2_PEP3	0.523
9	E3U93_14820/TFE48517.1	KXC18_14660/MBW2745566.1	helix-turn-helix domain-containing protein	ISMmy3_PEP	0.357
10	E3U93_05100/pseudogene	KXC18_06155/pseudogene	ISL3 family transposase	ISP1_PEP	0.833
11	E3U93_13645/TFE48946.1	KXC18_13665/MBW2745391.1	multidrug efflux SMR transporter	Integron_323714046	0.331
12	E3U93_12755/TFE49351.1	KXC18_11975/MBW2745065.1	helix-turn-helix domain-containing protein	ISLsa2_PEP	0.396
13	E3U93_12760/TFE49352.1	KXC18_11970/MBW2745064.1	hypothetical protein	ISLsa2_PEP3	0.507
14	E3U93_11995/pseudogene	KXC18_11585/pseudogene	IS30 family transposase	ISLsa1_PEP	0.678
15	E3U93_01615/TFE52687.1	KXC18_01560/MBW2743073.1	N-acetyltransferase	IS231K_PEP	0.232
16	E3U93_05100/pseudogene	KXC18_06155/pseudogene	ISL3 family transposase	ISP1_PEP	0.937
17	E3U93_11995/pseudogene	KXC18_11586/pseudogene	IS30 family transposase	ISSag3_PEP	0.372
18	E3U93_04110/TFE51597.1	KXC18_12600/MBW2745186.1	noncanonical pyrimidine nucleotidase, YjjG family	IS231K_PEP3	0.235
19	E3U93_06235/TFE51028.1	KXC18_07095/MBW2744141.1	HAD family hydrolase	IS231K_PEP3	0.261
20	E3U93_02060/pseudogene	KXC18_02005/pseudogene	IS30 family transposase	ISEnfa364_PEP4	0.394
21	E3U93_11980/TFE49422.1	KXC18_11575/MBW2744992.1	IS30 family transposase	IS1070_PEP	0.446
22	E3U93_01245/TFE52620.1	KXC18_01215/ MBW2743006.1	CPBP family intramembrane metalloprotease	IS231L_PEP	
[23]	E3U93_16150/ TFE47836.1	KXC18_15655/ MBW2745755.1	IS5-like element ISLpl3 family transposase	ISLpl3_PEP3	1.000
24	E3U93_11595/TFE49605.1		MerR family transcriptional regulator	ISPpu12_PEP	0.288
25	E3U93_13760/TFE48918.1		N-acetyltransferase	ISBce8_PEP2	0.290
26		JAHWFM010000022.1, 52573-52632		ISLpl3_PEP3	1.000
27		JAHWFM010000014.1, 2-61		ISLpl3_PEP3	1.000
28		KXC18_09780/MBW2744659.1	transposase (partial)	ISLpl3_PEP3	1.000
29		KXC18_09785/MBW2744660.1	transposase (partial)	ISP1_PEP	0.983
30		KXC18_09530/MBW2744611.1	MFS transporter	IS5564_PEP	0.257

[1]«Contig number, nucleotide positions» (if the region in the genome is not annotated) or «gene locus/protein ID» (if the region in the genome is annotated); [2]Protein names are given by strain 8p-a3. [ ] – a putative transposase that has moved in strain 8p-a3-Clr-Amx to the esterase gene.

Table S4. Proteins *L. plantarum* 8p-a3 and *L. plantarum* 8p-a3-Clr-Amx, whose genes are presumably of prophage origin (predicted using the VRprofile database)

№	Protein ID[1]	Protein[2]	Hit	Ha-value
1	TFE52395.1/MBW2742772.1	hypothetical protein	Prophage_148747763	0.386
2	TFE52398.1/MBW2742775.1	hypothetical protein	Prophage_23455787	0.605
3	TFE52399.1/MBW2742776.1	hypothetical protein	Prophage_157325474	0.492
4	TFE52747.1/MBW2742779.1	transketolase	Prophage_90592696	0.414
5	TFE52769.1/MBW2742780.1	transcriptional regulator	Prophage_23455797	0.342
6	TFE52403.1/MBW2742783.1	helix-turn-helix domain-containing protein	Prophage_13487829	0.335
7	TFE52404.1/MBW2742784.1	PBSX family phage terminase large subunit	Prophage_28876345	0.531
8	TFE52405.1/MBW2742785.1	phage portal protein	Prophage_16798786	0.368
9	TFE52406.1/MBW2742786.1	hypothetical protein	Prophage_157324964	0.322
10	TFE52407.1/MBW2742787.1	hypothetical protein	Prophage_28876340	0.230
11	TFE52408.1/MBW2742788.1	N4-gp56 family major capsid protein	Prophage_157324966	0.530
12	pseudo/MBW2742789.1	hypothetical protein ▲	Prophage_66395118	0.349
13	TFE52409.1/MBW2742790.1	hypothetical protein	Prophage_157324968	0.306
14	TFE52411.1/MBW2742793.1	hypothetical protein	Prophage_157324971	0.284
15	TFE52413.1/MBW2742794.1	hypothetical protein	Prophage_15088783	0.220
16	TFE52415.1/MBW2742796.1	hypothetical protein	Prophage_56694916	0.221
17	TFE52417.1/MBW2742798.1	phage tail family protein	Prophage_16798800	0.239
18	TFE52418.1/MBW2742799.1	hypothetical protein	Prophage_23455812	0.408
19	pseudo/MBW2742802.1	capsid protein ▲	Prophage_62327113	0.496
20	TFE52770.1/MBW2742807.1	LysM peptidoglycan-binding domain-containing protein	Prophage_48697278	0.283
21	TFE52426.1/MBW2742809.1	holin	Prophage_56693137	0.492
22	TFE52436.1/MBW2742818.1	class 1b ribonucleoside-diphosphate reductase subunit beta	Prophage_9630287	0.429
23	TFE52437.1/MBW2742819.1	class 1b ribonucleoside-diphosphate reductase subunit alpha	Prophage_48696468	0.384
24	TFE52438.1/MBW2742820.1	redoxin NrdH	Prophage_109302792	0.351
25	TFE52445.1/MBW2742827.1	dTMP kinase	Prophage_82701134	0.312
26	TFE52464.1/MBW2742845.1	co-chaperone GroES	Prophage_156564025	0.298
27	TFE52465.1/MBW2742846.1	chaperonin GroEL	Prophage_82701079	0.213
28	TFE52468.1/MBW2742849.1	phosphate ABC transporter substrate-binding protein	Prophage_61806371	0.247
29	TFE52515.1/MBW2742899.1	ATP-dependent Clp protease proteolytic subunit	Prophage_110804048	0.245
30	TFE52526.1/MBW2742910.1	ribonuclease R	Prophage_115304286	0.239
31	TFE52537.1/MBW2742921.1	exonuclease	Prophage_66395669	0.326
32	TFE52578.1/MBW2742964.1	alcohol dehydrogenase	Prophage_157325437	0.325
33	TFE52605.1/MBW2742991.1	NUDIX domain-containing protein	Prophage_48697515	0.290
34	TFE52618.1/MBW2743004.1	XRE family transcriptional regulator	Prophage_23455773	0.344

35	TFE52641.1/MBW2743027.1	site-specific integrase	Prophage_157325260	0.448
36	TFE52682.1/MBW2743067.1	cold-shock protein	Prophage_13095918	0.788
37	TFE52692.1/MBW2743078.1	deoxynucleoside kinase	Prophage_56693115	0.472
38	TFE51910.1/MBW2743181.1	alanine racemase	Prophage_80159713	0.308
39	TFE51937.1/MBW2743207.1	serine hydrolase	Prophage_38707787	0.218
40	TFE52070.1/MBW2743342.1	LysM domain-containing protein	Prophage_56693169	0.294
41	TFE52108.1/MBW2743380.1	nicotinamide mononucleotide transporter	Prophage_56693173	0.662
42	TFE52120.1 /MBW2743391.1	thioredoxin	Prophage_9630289	0.227
43	TFE52215.1/MBW2743490.1	NUDIX domain-containing protein	Prophage_38638625	0.263
44	TFE51628.1/MBW2745156.1	anaerobic ribonucleoside-triphosphate reductase activating protein	Prophage_157311352	0.324
45	TFE51787.1/MBW2745155.1	anaerobic ribonucleoside-triphosphate reductase	Prophage_38639964	0.428
46	TFE51704.1/MBW2744566.1	LysR family transcriptional regulator / Y-family DNA polymerase	Prophage_9630142	0.241
47	TFE51309.1/MBW2743913.1	fructose-6-phosphate aldolase	Prophage_113200706	0.493
48	TFE51437.1	CopY/TcrY family copper transport repressor ♦	Prophage_134287378	
49	TFE51454.1/MBW2743761.1	phosphatidylglycerophosphatase A	Prophage_56693170	0.476
50	TFE51014.1/MBW2744156.1	SHOCT domain-containing protein	Prophage_9633003	0.267
51	TFE51017.1/MBW2744154.1	glycosyltransferase	Prophage_46358650	0.403
52	TFE51068.1	bifunctional (p)ppGpp synthetase/guanosine-3',5'-bis(diphosphate) 3'-pyrophosphohydrolase ♦	Prophage_37651649	
53	TFE51072.1/MBW2744095.1	N-acetylmuramoyl-L-alanine amidase	Prophage_155042951	0.220
[54]	TFE51083.1/MBW2744084.1	PhoH family protein	Prophage_19343479	0.232
55	TFE51150.1/MBW2744017.1	DNA polymerase III subunit alpha	Prophage_29566684	0.263
56	TFE51167.1/MBW2744000.1	HU family DNA-binding protein	Prophage_156564019	0.681
57	TFE51174.1/MBW2743993.1	thymidylate synthase	Prophage_148747783	0.497
58	TFE51198.1/MBW2743970.1	DNA topoisomerase IV subunit B	Prophage_116326225	0.271
59	TFE51199.1/MBW2743969.1	DNA topoisomerase IV subunit A	Prophage_80159786	0.239
60	TFE50718.1/MBW2744355.1	2-deoxyuridine 5-triphosphate nucleotidohydrolase / dUTPase	Prophage_155042924	0.251
61	TFE50724.1/MBW2744349.1	ribosomal-processing cysteine protease Prp	Prophage_41179241	0.288
62	TFE50807.1/MBW2744264.1	site-specific integrase	Prophage_155042957	0.329
63	TFE50808.1/MBW2744263.1	hypothetical protein / phage integrase SAM-like domain-containing protein	Prophage_134287379	0.255
64	TFE50812.1/MBW2744260.1	hypothetical protein	Prophage_9633063	0.478
65	TFE50870.1/MBW2745413.1	glycosyl hydrolase family 25	Prophage_56693136	0.252
66	TFE50464.1/MBW2744514.1	transposase	Prophage_119443708	0.362
67	pseudo	IS1182 family transposase	Prophage_9635722	0.406
68	TFE50509.1	ribokinase ♦	Prophage_61806181	
69	TFE50518.1/MBW2744462.1	helix-turn-helix domain-containing protein	Prophage_17426234	0.243
70	TFE50533.1/MBW2744446.1	DNA polymerase III subunit beta	Prophage_156564023	0.235
71	TFE50539.1/MBW2744440.1	single-stranded DNA-binding protein	Prophage_15088755	0.525
72	TFE50543.1/MBW2744436.1	replicative DNA helicase	Prophage_28876155	0.511
73	TFE50559.1/MBW2744420.1	site-specific integrase	Prophage_28876262	0.359

74	TFE50564.1/MBW2744416.1	DNA replication protein / bifunctional DNA primase/polymerase	Prophage_13095885	0.280
75	TFE50565.1/MBW2744415.1	virulence protein	Prophage_66395453	0.345
76	TFE50568.1/MBW2744412.1	head-tail adaptor protein / phage head closure protein	Prophage_13095850	0.330
77	TFE50569.1/MBW2744411.1	HNH endonuclease	Prophage_119443688	0.357
78	TFE50570.1/MBW2744410.1	phage terminase small subunit P27 family	Prophage_28876231	0.287
79	TFE50571.1/MBW2744409.1	terminase large subunit	Prophage_28876230	0.372
80	TFE50573.1/MBW2744407.1	phage portal protein	Prophage_28876227	0.285
81	TFE50575.1/MBW2744405.1	phage gp6-like head-tail connector protein / head-tail connector protein	Prophage_9632901	0.362
82	TFE50576.1/MBW2744404.1	hypothetical protein	Prophage_78000018	0.213
83	TFE50583.1/MBW2744398.1	MBL fold metallo-hydrolase	Prophage_157325335	0.218
84	TFE50276.1/MBW2743541.1	XRE family transcriptional regulator	Prophage_28876313	0.210
85	TFE50377.1/MBW2743637.1	site-specific integrase	Prophage_13095806	0.279
86	TFE50378.1	deoxyribonuclease ♦	Prophage_28876265	
87	TFE50382.1/MBW2743643.1	ImmA/IrrE family metallo-endopeptidase	Prophage_155042958	0.291
88	TFE50383.1/MBW2743644.1	helix-turn-helix domain-containing protein	Prophage_157325325	0.458
89	TFE50384.1/MBW2743645.1	XRE family transcriptional regulator	Prophage_157325326	0.342
90	TFE50143.1/MBW2744748.1	site-specific integrase	Prophage_22296542	0.339
91	TFE50192.1/MBW2744745.1	XRE family transcriptional regulator	Prophage_28876431	0.610
92	TFE50148.1/MBW2744744.1	phage regulatory protein / Rha family transcriptional regulator	Prophage_78000000	0.241
93	TFE50149.1/MBW2744743.1	DUF771 domain-containing protein	Prophage_66395845	0.303
94	TFE50152.1/MBW2744738.1	hypothetical protein / host-nuclease inhibitor Gam family protein	Prophage_122891799	0.341
95	TFE50153.1/MBW2744737.1	nucleotide-binding protein / AAA family ATPase	Prophage_23455783	0.909
96	TFE50154.1/MBW2744736.1	DUF669 domain-containing protein	Prophage_23455784	0.745
97	TFE50155.1/MBW2744735.1	hypothetical protein	Prophage_23455785	0.839
98	TFE50156.1/MBW2744734.1	replication protein	Prophage_41179305	0.404
99	TFE50157.1/MBW2744733.1	ATP-binding protein	Prophage_48697291	0.452
100	TFE50159.1/MBW2744730.1	hypothetical protein	Prophage_23455792	0.731
101	TFE50161.1/MBW2744728.1	hypothetical protein / YopX family protein	Prophage_23455793	0.479
102	TFE50162.1/MBW2744727.1	DUF1642 domain-containing protein	Prophage_56693057	0.376
103	TFE50164.1/MBW2744725.1	transcriptional regulator	Prophage_66395320	0.255
104	TFE50193.1/MBW2744722.1	HNH endonuclease	Prophage_9633038	0.482
105	TFE50166.1/MBW2744721.1	phage terminase small subunit P27 family	Prophage_13095783	0.566
106	TFE50167.1/MBW2744720.1	terminase large subunit	Prophage_14251127	0.647
107	TFE50169.1/MBW2744718.1	phage portal protein	Prophage_9633041	0.525
108	TFE50170.1/MBW2744717.1	Clp protease ClpP	Prophage_9633042	0.473
109	TFE50171.1/MBW2744716.1	phage major capsid protein	Prophage_9634653	0.416
110	TFE50172.1/MBW2744715.1	phage gp6-like head-tail connector protein / head-tail connector protein	Prophage_9632425	0.393
111	TFE50176.1/MBW2744711.1	phage tail protein	Prophage_48697269	0.443
112	TFE50180.1/MBW2744707.1	phage tail protein	Prophage_148750855	0.299

113	TFE50181.1/MBW2744706.1	hypothetical protein / phage tail protein	Prophage_148750856	0.370
114	TFE49889.1/MBW2744893.1	glycosyltransferase / glycosyltransferase family 2 protein	Prophage_19549012	0.363
115	TFE49979.1/MBW2744514.1	transposase	Prophage_119443708	0.362
116	TFE49738.1/MBW2743722.1	membrane protein insertion efficiency factor YidD	Prophage_9628603	0.247
117	TFE49750.1/MBW2743710.1	uracil permease / NCS2 family nucleobase:cation symporter	Prophage_80159777	0.228
118	TFE49753.1/MBW2743707.0	serine hydroxymethyltransferase	Prophage_66391812	0.488
119	TFE49804.1/MBW2743703.1	thymidine kinase	Prophage_116326329	0.484
120	pseudo/MBW2743686.1	LysM peptidoglycan-binding domain-containing protein ▲	Prophage_78000035	0.443
121	TFE49805.1/MBW2743683.1	LysM peptidoglycan-binding domain-containing protein	Prophage_41179337	0.242
122	TFE49777.1/MBW2743679.1	hypothetical protein	Prophage_41179261	0.583
123	TFE49778.1/MBW2743678.1	phage tail protein	Prophage_41179260	0.415
124	TFE49807.1/MBW2743677.1	hypothetical protein	Prophage_41179259	0.447
125	TFE49779.1/MBW2743676.1	hypothetical protein	Prophage_41179250	0.614
126	TFE49808.1/MBW2743675.1	phage tail protein	Prophage_41179249	0.583
127	TFE49780.1/MBW2743674.1	hypothetical protein	Prophage_41179248	0.521
128	TFE49781.1/MBW2743673.1	HK97 gp10 family phage protein	Prophage_41179258	0.557
129	TFE49782.1/MBW2743672.1	hypothetical protein	Prophage_41179247	0.559
130	TFE49783.1/MBW2743671.1	hypothetical protein	Prophage_41179246	0.318
131	TFE49785.1/MBW2743669.1	major capsid protein / phage head-tail connector protein	Prophage_41179245	0.509
132	TFE49786.1/MBW2743668.1	hypothetical protein	Prophage_41179244	0.448
133	TFE49787.1/MBW2743667.1	DUF4355 domain-containing protein	Prophage_41179243	0.347
134	TFE49790.1/MBW2743664.1	phage head morphogenesis protein / minor capsid protein	Prophage_41179242	0.298
135	TFE49792.1/MBW2743662.1	phage portal protein	Prophage_41179240	0.466
136	TFE49793.1/MBW2743661.1	PBSX family phage terminase large subunit	Prophage_122891825	0.551
137	TFE49794.1/MBW2743660.1	terminase small subunit	Prophage_23455798	0.680
138	TFE49809.1/MBW2743659.1	DUF2829 domain-containing protein	Prophage_34419530	0.266
139	TFE49799.1/MBW2743654.1	oxidoreductase / phage antirepressor KilAC domain-containing protein	Prophage_29028674	0.458
140	TFE49588.1/MBW2744972.1	XRE family transcriptional regulator / helix-turn-helix domain-containing protein	Prophage_23505482	0.226
141	TFE49608.1/MBW2744951.1	glucose-6-phosphate dehydrogenase	Prophage_113200631	0.314
142	TFE49637.1/MBW2744923.1	bifunctional phosphoribosylaminoimidazolecarboxamide formyltransferase/IMP cyclohydrolase	Prophage_61806062	0.276
143	TFE49638.1	phosphoribosylglycinamide formyltransferase ♦	Prophage_61805923	0.302
144	TFE49639.1/MBW2744921.1	phosphoribosylformylglycinamidine cyclo-ligase	Prophage_61806048	0.384
145	TFE49641.1/MBW2744919.1	phosphoribosylformylglycinamidine synthase subunit PurL	Prophage_149882852	0.395
146	TFE49411.1/MBW2744980.1	site-specific integrase	Prophage_41179288	0.337
147	TFE49413.1/MBW2744983.1	phage regulatory protein / Rha family transcriptional regulator	Prophage_13095661	0.232
148	TFE49417.1/MBW2744987.1	DNA replication protein / bifunctional DNA primase/polymerase	Prophage_9632925	0.302
149	TFE49418.1/MBW2744988.1	virulence protein / virulence-associated E family protein	Prophage_66395590	0.288
150	TFE49428.1/MBW2744997.1	serine-threonine protein phosphatase / metallophosphoesterase	Prophage_82547740	0.261
151	TFE49430.1/MBW2744999.1	ribosyl nicotinamide transporter	Prophage_56693131	0.374

152	TFE49433.1/MBW2745033.1	GntR family transcriptional regulator	Prophage_40807292	0.388
153	TFE49463.1	dUTP diphosphatase ♦	Prophage_80159764	
154	TFE49482.1/MBW2745050.1	integrase / tyrosine-type recombinase/integrase	Prophage_157325322	0.275
155	TFE49484.1/MBW2745052.1	hypothetical protein / ImmA/IrrE family metallo-endopeptidase	Prophage_23455774	0.937
156	TFE49486.1/MBW2745054.1	XRE family transcriptional regulator	Prophage_23455778	0.977
157	TFE49487.1/MBW2745055.1	DUF2513 domain-containing protein	Prophage_28876256	0.323
158	TFE49489.1/MBW2745059.1	hypothetical protein	Prophage_23455781	1.000
159	TFE49307.1/MBW2745111.1	ComE operon protein 2	Prophage_156564196	0.422
160	TFE49320.1/MBW2745098.1	nucleotide pyrophosphohydrolase	Prophage_15320596	0.333
161	TFE49342.1/MBW2745074.1	single-stranded-DNA-specific exonuclease RecJ	Prophage_156564062	0.228
162	TFE49349.1/MBW2745067.1	site-specific integrase	Prophage_48697280	0.330
163	TFE49163.1/MBW2745210.1	septum site-determining protein MinC	Prophage_22091150	0.257
164	TFE49175.1/MBW2745221.1	recombinase RecA	Prophage_109302868	0.487
165	TFE49224.1/MBW2745270.1	metallophosphatase	Prophage_157325188	0.317
166	TFE49053.1/MBW2745287.1	UDP-galactopyranose mutase	Prophage_109290213	0.332
167	TFE49086.1/MBW2745299.1	integrase / tyrosine-type recombinase/integrase	Prophage_31415840	0.380
168	TFE49087.1/MBW2745298.1	dTDP-glucose 4,6-dehydratase	Prophage_61806141	0.257
169	*TFE49091.1/MBW2745294.1*	*hypothetical protein / sugar transferase*	*Prophage_56693090*	*0.249*
170	TFE48885.1/MBW2745403.1	GTP cyclohydrolase I FolE	Prophage_146329945	0.390
171	TFE48904.1/MBW2745381.1	GMP reductase	Prophage_66395520	0.708
172	TFE48905.1/MBW2745380.1	adenylosuccinate synthase	Prophage_109393431	0.242
173	TFE48638.1	DNA translocase FtsK ♦	Prophage_29566938	0.214
174	TFE48657.1/MBW2745509.1	NUDIX hydrolase	Prophage_56693134	0.239
175	TFE48670.1/MBW2745522.1	ATP-dependent RecD-like DNA helicase	Prophage_80159748	0.217
176	TFE48426.1/MBW2745603.1	cell wall hydrolase / glycoside hydrolase family 73 protein	Prophage_22855012	0.305
177	TFE48430.1	DNA helicase PcrA ♦	Prophage_156564011	0.370
178	TFE48431.1/MBW2745598.1	NAD-dependent DNA ligase LigA	Prophage_80159718	0.339
179	TFE48219.1/MBW2745694.1	decarboxylating 6-phosphogluconate dehydrogenase	Prophage_113200632	0.242
180	TFE48222.1/MBW2745691.1	glycerol-3-phosphate cytidylyltransferase	Prophage_61806180	0.356
181	TFE48227.1/MBW2745686.1	XRE family transcriptional regulator	Prophage_9633005	0.288
182	TFE48228.1	gamma-D-glutamyl-meso-diaminopimelate peptidase ♦	Prophage_134287357	
183	*TFE48029.1/MBW2745721.1*	*SGNH/GDSL hydrolase family protein*	*Prophage_41179332*	*0.282*
184	TFE48035.1	pyruvate formate lyase-activating protein ♦	Prophage_38707878	
185	MBW2743911.1	sugar-phosphatase ▲	Prophage_115304277	0.221
186	MBW2745634.1	flavodoxin ▲	Prophage_156564204	0.223
187	MBW2745657.1	DUF2829 domain-containing protein ▲	Prophage_157325209	0.352
188	MBW2745659.1	HIT family protein ▲	Prophage_109393233	0.254
189	TFE52474.1	peptide chain release factor	Prophage_148912865	
190	TFE52583.1	transcriptional repressor	Prophage_23752328	

191	TFE52618.1	XRE family transcriptional regulator	Prophage_115334658	
192	TFE52052.1	XRE family transcriptional regulator	Prophage_31415823	
193	TFE52071.1	LysM domain-containing protein	Prophage_115315596	
194	TFE52215.1	NUDIX domain-containing protein	Prophage_66391637	
[195]	TFE51695.1	LysM peptidoglycan-binding domain-containing protein	Prophage_9630590	
196	TFE51089.1	DNA primase	Prophage_118197643	
197	TFE50847.1	diguanylate cyclase	Prophage_30043986	
198	TFE50860.1	Holliday junction resolvase RecU	Prophage_115334618	
199	TFE50377.1	site-specific integrase	Prophage_13095681	
200	TFE49727.1	glycerophosphodiester phosphodiesterase	Prophage_48696447	
201	TFE49053.1	UDP-galactopyranose mutase	Prophage_30044114	
202	TFE49097.1	UDP-galactopyranose mutase	Prophage_30044114	
203	TFE48443.1	lysozyme	Prophage_110804057	
204	TFE48447.1	DEAD/DEAH box helicase	Prophage_157325275	

[1]Protein ID of 8p-a3 strain / Protein ID of 8p-a3-Clr-Amx strain; [2]If the protein names of the strains differ, then both are listed in the order 8p-a3/8p-a3-Clr-Amx; ♦ - the protein is predicted only for strain 8p-a3; ▲ - the protein is predicted only for strain 8p-a3-Clr-Amx; proteins whose genes have different lengths in strains are highlighted in *italics*; [ ] – a mutation was found in the corresponding *L. plantarum* 8p-a3-Clr-Amx gene.

Table S5. Virulence factors detected *in silico* in *L. plantarum* strains 8p-a3 and 8p-a3-Clr-Amx (based on VFDB database)

Protein of *L. plantarum* 8p-a3	Gene locus	Bacterial virulence factor
**Adhesion**		
Chaperonin GroEL	TFE52465.1	GroEL (Clostridium)
Bifunctional acetaldehyde-CoA/alcohol dehydrogenase	TFE50511.1	Listeria adhesion protein (Listeria)
Type I glyceraldehyde-3-phosphate dehydrogenase	TFE52518.1	Streptococcal plasmin receptor/GAPDH (Streptococcus)
**Other proteins involved in adhesion**		
Elongation factor Tu	TFE49316.1	EF-Tu (Mycoplasma)
**Antiphagocytosis**		
Isoprenyl transferase	TFE51033.1	Capsule (Enterococcus)
UDP-galactopyranose mutase	TFE49053.1; TFE49097.1	Capsule (Enterococcus)
ABC transporter ATP-binding protein	TFE52027.1	Capsule (Enterococcus)
NADP-dependent phosphogluconate dehydrogenase	TFE49957.1	Capsule (Klebsiella)
**Invasion**		
GtrA family protein	TFE48389.1	Cell wall teichoic acid glycosylation protein (Listeria)
**Proteases**		
PDZ domain-containing protein	TFE50584.1	Serine protease (Streptococcus)
Trigger factor	TFE49317.1	Trigger factor (Streptococcus)
**Secretion systems**		
ATP-dependent chaperone ClpB	TFE51148.1	T6SS-II (Klebsiella)
**Toxins**		
TlyA family rRNA (cytidine-2'-O)-methyltransferase	TFE50734.1	Hemolysin (Clostridium)
Adenylyl-sulfate kinase	TFE48261.1	Phytotoxin phaseolotoxin (Pseudomonas)
**Resistance to bile**		
Choloylglycine hydrolase family protein	TFE51312.1	Bile-salt hydrolase (Listeria)
**Components on the cell surface**		
sn-glycerol-3-phosphate ABC transporter ATP-binding protein UgpC	TFE50486.1	Trehalose-recycling ABC transporter (Mycobacterium)
**Enzymes**		
Phosphopyruvate hydratase	TFE49885.1; TFE51133.1; TFE52521.1	Streptococcal enolase (Streptococcus)
**Immunological evasion**		
UDP-glucose 4-epimerase GalE	TFE51360.1; TFE52451.1	Polysaccharide capsule (Bacillus)
Flippase	TFE49089.1	Capsule (Streptococcus)
UDP-N-acetylglucosamine 2-epimerase (non-hydrolyzing)	TFE49100.1	Capsule (Streptococcus)
LysR family transcriptional regulator	TFE51185.1	Capsule (Streptococcus)
UTP-glucose-1-phosphate uridylyltransferase GalU	TFE52488.1	Capsule (Streptococcus)
dTDP-glucose 4,6-dehydratase	TFE49087.1	Capsule (Streptococcus)
Sugar transferase	TFE48237.1	Polysaccharide capsule (Bacillus)
**Absorption of manganese**		
Metal ABC transporter substrate-binding protein	TFE52756.1	Pneumococcal surface antigen A / Metal binding protein SloC (Streptococcus)
**Arrest of phagosome maturation**		
Nucleoside-diphosphate kinase	TFE52116.1	Nucleoside diphosphate kinase (Mycobacterium)
**Regulation**		

Response regulator transcription factor	TFE49959.1	LisR/LisK (Listeria)
RNA polymerase sigma factor RpoD	TFE51090.1	Sigma A (Mycobacterium)
**Adaptation to stress**		
Catalase	TFE51461.1	Catalase (Neisseria)
**Attachment of proteins to the cell surface**		
Prolipoprotein diacylglyceryl transferase	TFE52486.1	Lipoprotein diacylglyceryl transferase (Listeria)

Table S6. Mobile genetic elements (MGE) of *L. plantarum* 8p-a3 and *L. plantarum* 8p-a3-Clr-Amx associated with virulence factors (predicted using the VRprofile database)

№	Protein of *L. plantarum*[1]	Gene locus / Protein ID[2]	MGE	Origin
1	peptide chain release factor 2	E3U93_00450 / TFE52474.1	Prophage_148912865	Pseudomonas phage 73
2	transcriptional repressor	E3U93_01045 / TFE52583.1	Prophage_23752328	Burkholderia phage Bcep781
3	XRE family transcriptional regulator	E3U93_01235 / TFE52618.1	Prophage_115334658	Geobacillus phage GBSV1
4	CPBP family intramembrane metalloprotease	E3U93_01245 / TFE52620.1	IS231L_PEP	no data
5	N-acetyltransferase	E3U93_01615 / TFE52687.1	IS231K_PEP	no data
6	Cof-type HAD-IIB family hydrolase	E3U93_02695 / TFE51994.1	Prophage_115304277	Lactococcus phage Q54
7	XRE family transcriptional regulator	E3U93_03000 / TFE52052.1	Prophage_31415823	Bacillus phage phBC6A52
8	LysM domain-containing protein	E3U93_03095 / TFE52071.1	Prophage_115315596	Lactococcus phage 712
9	NUDIX domain-containing protein	E3U93_03845 / TFE52215.1	Prophage_66391637	Enterobacteria phage RB43
10	LysM peptidoglycan-binding domain-containing protein	E3U93_04615 / TFE51695.1	Prophage_9630590	Lactococcus phage bIL170
11	Y-family DNA polymerase	E3U93_04655 / TFE51703.1	Prophage_9630142	Bacillus phage SPBc2
12	CopY/TcrY family copper transport repressor	E3U93_06030 / TFE51437.1	Prophage_134287378	Clostridium phage phiC2
13	phosphatidylglycerophosphatase A	E3U93_06120 / TFE51454.1	Prophage_56693170	Lactobacillus phage LP65
14	DNA primase	E3U93_06555 / TFE51089.1	Prophage_118197643	Thermus phage phiYS40
15	DNA polymerase III subunit alpha	E3U93_06870 / TFE51150.1	Prophage_29566684	Mycobacterium phage Bxz1
16	diguanylate cyclase	E3U93_07950 / TFE50847.1	Prophage_30043986	Staphylococcus phage phiN315
17	Holliday junction resolvase RecU	E3U93_08020 / TFE50860.1	Prophage_115334618	Geobacillus phage GBSV1
18	ribokinase	E3U93_08590 / TFE50509.1	Prophage_61806181	Cyanophage P-SSM2
19	site-specific integrase	E3U93_09750 / TFE50377.1	Prophage_13095681	Lactococcus phage bIL285
20	glycerophosphodiester phosphodiesterase	E3U93_11040 / TFE49727.1	Prophage_48696447	Staphylococcus phage K
21	MerR family transcriptional regulator	E3U93_11595 / TFE49605.1	ISPpu12_PEP	no data
22	glucose-6-phosphate dehydrogenase	E3U93_11610 / TFE49608.1	Prophage_113200631	Phage Syn9
23	helicase-exonuclease AddAB subunit AddA	E3U93_11660 / TFE49617.1	Prophage_156564011	Bacillus phage 0305phi8-36
24	phosphoribosylformylglycinamidine synthase subunit PurL	E3U93_11780 / TFE49641.1	Prophage_149882852	Microbacterium phage Min1
25	GntR family transcriptional regulator	E3U93_12045 / TFE49433.1	Prophage_40807292	Streptomyces phage phiC31
26	thioredoxin	E3U93_13000 / TFE49198.1	Prophage_9630289	Bacillus phage SPBc2
27	ribokinase	E3U93_13085 / TFE49215.1	Prophage_61806181	Cyanophage P-SSM2
28	UDP-galactopyranose mutase	E3U93_13225 / TFE49053.1	Prophage_30044114	Rhodothermus phage RM378
29	UDP-galactopyranose mutase	E3U93_13490 / TFE49097.1	Prophage_30044114	Rhodothermus phage RM378
30	LysM domain-containing protein	E3U93_14060 / TFE48802.1	Prophage_56693169	Lactobacillus phage LP65
31	lysozyme	E3U93_15105 / TFE48443.1	Prophage_110804057	Clostridium perfringens phage phiSM101
32	DEAD/DEAH box helicase	E3U93_15125 / TFE48447.1	Prophage_157325275	Listeria phage B025

[1] - the lists of virulence factors predicted using the algorithms of the VRprofile and VFDB databases differ; [2] – the numbers for strain 8p-a3 are indicated; [ ] – a change in the primary DNA structure was detected in the corresponding locus of *L. plantarum* 8p-a3-Clr-Amx

Table S7. The effect of *D. melanogaster* infection by different strains of *L. plantarum* on the reproduction and viability of flies

Strain of *L. plantarum*	Virulence parameters	DF	SS	MS	F	**p**	**η^2**
**8p-a3**	Egg production	1	435.6	435.6	15.92	0.004	0.66
	Embryonic death	1	40.0	40.0	6.15	0.03	0.43
	Egg production index	1	0.06	0.06	103.32	0.000007	0.93
	DNA damage index	1	2.2	2.2	14.82	0.005	0.65
	Intestinal tissue damage	1	2496.4	2496.4	123.58	0.000004	0.94
**8p-a3-Clr-Amx**	Egg production	1	1968.3	1968.3	57.87	0.000003	0.82
	Embryonic death	1	260.3	260.3	9.24	0.01	0.44
	Egg production index	1	0.31	0.31	182.09	0.000001	0.95
	DNA damage index	1	7.39	7.39	30.18	0.0005	0.79
	Intestinal tissue damage	1	6864.4	6864.4	409.81	0.000000	0.99
**AG1**	Egg production	1	506.9	506.9	22.41	0.001	0.74
	Embryonic death	1	775.9	775.9	66.69	0.00003	0.89
	Egg production index	1	0.07	0.07	1059.84	0.000000	0.99
	DNA damage index	1	3.2	3.2	17.9	0.002	0.69
	Intestinal tissue	1	4977.3	4977.3	224.1	0.000000	0.9

	damage						6
**AG10**	Egg production	1	280.9	280.9	19.85	0.002	0.71
	Embryonic death	1	217.1	217.1	15.81	0.004	0.66
	Egg production index	1	0.25	0.25	5.68	0.04	0.41
	DNA damage index	1	0.16	0.16	1.71	0.22	0.17
	Intestinal tissue damage	1	6.4	6.4	0.71	0.42	0.08
**DMC-S1**	Egg production	1	84.1	84.1	0.37	0.54	0.02
	Embryonic death	1	39.3	39.3	2.49	0.13	0.12
	Egg production index	1	0.09	0.09	2.15	0.18	0.21
	DNA damage index	1	0.32	0.32	2.27	0.17	0.22
	Intestinal tissue damage	1	22.5	22.5	1.04	0.33	0.11

DF – the degrees of freedom in the source; SS – the sum of squares due to the source; MS – the mean sum of squares due to the source; F – the *F*-statistics; p- the *p*-value; η^2 – the partial eta-squared (expresses the degree of influence or the strength of the effect (factor) on the dependent variable (attribute); the value of η^2 lies in the range from 0 to 1).whether the strength of the effect (factor) on the dependent variable (attribute); the value of η^2 lies in the range from 0 to 1).

Table S8. Comparative analysis of survival curves and relative mortality risk of drosophila infected with different strains of *L. plantarum*

Strain	Sex	χ^2	p	RR	95% CI	p
L. plantarum 8p-a3	Females	7.675	0.0056	2.9444	1.8639 - 4.6514	< 0.0001
	Males	13.66	0.0002	3.8889	1.9740 - 7.6614	0.0001
L. plantarum 8p-a3-Clr-Amx	Females	16.70	0.0001	3.8333	2.4726 - 5.9430	< 0.0001
	Males	42.01	0.0001	5.6667	2.9518 - 10.8784	< 0.0001
L. plantarum AG1	Females	2.805	0.0940	2.3333	1.4474 - 3.7616	0.0005
	Males	7.837	0.0051	3.0000	1.4873 - 6.0512	0.0021
L. plantarum AG10	Females	4.728	0.0297	2.6667	1.6743 - 4.2472	< 0.0001
	Males	4.436	0.0352	1.6667	0.7651 - 3.6305	0.1984
L. plantarum DMC-S1	Females	3.973	0.0462	0.3889	0.1699 - 0.8900	0.0254
	Males	5.825	0.0158	0.5556	0.1930 - 1.5996	0.2760

χ^2 – χ^2 log-rank (Mantel-Cox) test, using Graphpad Prism version 6.0; RR – Relative risk of mortality; 95% CI – 95% confidence interval.

The relative risk of mortality shows the probability of death of a part of the population against the risk of mortality for the remaining groups. An RR value below 1.0 indicates a lower risk of death under the influence of the factor in the study group than in all other groups, and above 1.0 – vice versa. A significant change is considered at $p < 0.05$.

Table S9. Parameters of the specific growth rate and generation time, maximum specific growth rate and lag time of *L. plantarum* 8p-a3, AG1, AG10, DMC-S1 and 8p-a3-Clr-Amx when cultivating bacteria on the MRS nutrient medium

#	Strain	**μ, h^{-1}**	**Generation time, h**	**$μ_{max}$, h^{-1}**	**Lag time, h**
1	*L. plantarum* 8p-a3	0.745 ± 0.035[2,3,4,5]	0.932 ± 0.044[2,3,4,5]	0.949 ± 0.027[2,3,5]	3.6 ± 0.2
2	*L. plantarum* 8p-a3-Clr-Amx	0.387 ± 0.01[1,3,4,5]	1.792 ± 0.046[1,3,4,5]	0.581 ± 0.009[1,3,4,5]	10 ± 0.4
3	*L. plantarum* AG1	0.503 ± 0.042[1,2,4]	1.385 ± 0.111[1,2,4]	0.791 ± 0.053[1,2,4,5]	5.2 ± 0.3
4	*L. plantarum* AG10	0.59 ± 0.032[1,2,3,5]	1.177 ± 0.064[1,2,3,5]	0.958 ± 0.061[2,3]	4.8 ± 0.2
5	*L. plantarum* DMC-S1	0.469 ± 0.01[1,2,4]	1.478 ± 0.031[1,2,4]	1.029 ± 0.034[1,2,3]	6.5 ± 0.2

μ - specific growth rate; $μ_{max}$ – maximum specific growth rate.

The data is presented in the form of averages and standard deviation. The data was compared using Tukey's multiple comparisons test using Ordinary one-way ANOVA. All strains were compared with each other in pairs: [1] - significant differences compared to *L. plantarum* 8p-a3 ($p = 0.0001$), [2] - significant differences compared to *L. plantarum* 8p-a3-Clr-Amx ($p = 0.0001$), [3] - significant differences compared to *L. plantarum* AG1 ($p = 0.0001$), [4] - significant differences compared to *L. plantarum* AG10 ($p = 0.0001$), [5] - significant differences compared to *L. plantarum* DMC-S1 ($p = 0.0001$).
